# SLX-1 Is Required for Maintaining Genomic Integrity and Promoting Meiotic Noncrossovers in the *Caenorhabditis elegans* Germline

**DOI:** 10.1371/journal.pgen.1002888

**Published:** 2012-08-23

**Authors:** Takamune T. Saito, Firaz Mohideen, Katherine Meyer, J. Wade Harper, Monica P. Colaiácovo

**Affiliations:** 1 Department of Genetics, Harvard Medical School, Boston, Massachusetts, United States of America; 2 Departments of Pathology and Cell Biology, Harvard Medical School, Boston, Massachusetts, United States of America; Baylor College of Medicine, United States of America

## Abstract

Although the SLX4 complex, which includes structure-specific nucleases such as XPF, MUS81, and SLX1, plays important roles in the repair of several kinds of DNA damage, the function of SLX1 in the germline remains unknown. Here we characterized the endonuclease activities of the *Caenorhabditis elegans* SLX-1-HIM-18/SLX-4 complex co-purified from human 293T cells and determined SLX-1 germline function via analysis of *slx-1*(*tm2644*) mutants. SLX-1 shows a HIM-18/SLX-4–dependent endonuclease activity toward replication forks, 5′-flaps, and Holliday junctions. *slx-1* mutants exhibit hypersensitivity to UV, nitrogen mustard, and camptothecin, but not gamma irradiation. Consistent with a role in DNA repair, recombination intermediates accumulate in both mitotic and meiotic germ cells in *slx-1* mutants. Importantly, meiotic crossover distribution, but not crossover frequency, is altered on chromosomes in *slx-1* mutants compared to wild type. This alteration is not due to changes in either the levels or distribution of double-strand breaks (DSBs) along chromosomes. We propose that SLX-1 is required for repair at stalled or collapsed replication forks, interstrand crosslink repair, and nucleotide excision repair during mitosis. Moreover, we hypothesize that SLX-1 regulates the crossover landscape during meiosis by acting as a noncrossover-promoting factor in a subset of DSBs.

## Author Summary

Crossover formation between homologous chromosomes is important for generating genetic diversity in subsequent generations, as well as for promoting accurate chromosome segregation during meiosis, which is a specialized cell division program that results in the formation of haploid gametes (sperm and eggs) from diploid parental germ cells. In the nematode *Caenorhabditis elegans*, a single off-centered crossover is formed on the chromosome arms between every pair of homologous chromosomes. Crossover formation at the central region of the chromosomes is suppressed by unknown mechanisms. By using high-resolution 3-D microscopy, we found that, while crossover distribution is biased to the arm regions along the chromosomes, DNA double-strand breaks (DSBs), which initiate the homologous recombination repair process, are evenly distributed along the chromosomes. These results suggest the existence of mechanisms that inhibit crossover formation after induction of DSBs at the central region of the chromosomes. In this study, our findings lead us to hypothesize that SLX-1, a structure-specific endonuclease, inhibits crossover formation at the central region of the chromosomes, probably via its resolution activity of the Holliday junctions, which are four-stranded recombination intermediates, to produce noncrossover products.

## Introduction

Genomic DNA is subjected to a variety of endogenous and exogenous sources of damage. To repair this DNA damage, the structure-specific endonucleases function to cleave branched DNA structures such as Y forks, 5′-flaps, 3′-flaps, stem loops, bubbles, replication forks (RFs) and Holliday junctions (HJs). SLX-1, a structure-specific nuclease that is highly conserved in eukaryotes, harbors both GIY-YIG-type endonuclease and PHD-type zinc finger domains. Furthermore, SLX1 requires the regulatory subunit SLX4 to perform its nuclease activities in both yeast and humans [1]–[5]. Specifically, the budding yeast Slx1-Slx4 complex, co-purified from *Escherichia coli*, shows endonuclease activity towards Y forks, 5′-flaps, RFs and HJs [1]. The immunoprecipitation products of fission yeast Slx1-TAP cleave stem loops and HJs [2]. Finally, the human SLX1-SLX4 complex, purified from both human cells and *E. coli*, cleaves Y forks, 3′-flaps, 5′-flaps, RFs, stem loops and HJs [3]–[5]. However, it is not known whether these endonuclease activities of SLX1 are conserved in other species such as flies and worms.

*slx1* was first identified in a synthetic-lethal screen for genes required for the viability of cells lacking Sgs1, the budding yeast RecQ helicase [6]. Sgs1 functions as an anti-recombinase by unwinding and dissolving toxic recombination intermediates, thereby maintaining genome stability [7]. *slx1* deletion (*slx1Δ*) mutants do not exhibit lethality, DNA damage sensitivity or sterility in either budding or fission yeast [2], [6], [8]. However, they exhibit defects in the completion of rDNA replication [2], [9], [10]. Moreover, in humans, the siRNA based-depletion of SLX1 increases both the endogenous and exogenous levels of DNA damage resulting from exposure to ionizing radiation (IR), camptothecin (CPT) and DNA interstrand crosslinking agents [3]–[5], [11]. In humans, SLX4 forms a complex with three structure-specific nucleases, SLX1, XPF-ERCC1 and MUS81-EME1 [3]–[5]. Moreover, individuals carrying mutations in *SLX4* exhibit symptoms of Fanconi anemia, a syndrome characterized by chromosomal instability in humans [12]. A mouse knockout of Slx4 also shows chromosomal instability phenotypes similar to those of Fanconi anemia in humans [13]. In budding yeast, Slx4 binds to Slx1 and Rad1^XPF^ in a mutually exclusive manner [6], [14], [15]. We showed that HIM-18, the SLX4 homolog in *C. elegans*, interacts with SLX-1 and XPF-1 [16]. Furthermore, we found that HIM-18/SLX-4 (referred to herein as HIM-18) and XPF-1 are required for wild type levels of meiotic crossover formation [16]. However, whether SLX-1 is required for meiotic crossover formation remains unknown. Moreover, whether SLX1, XPF and MUS81 function as structure-specific endonucleases either in the same or in different complexes *in vivo* is also unclear.

Here we show that *Caenorhabditis elegans* SLX-1 cleaves branched DNA substrates in a HIM-18/SLX-4-dependent manner *in vitro*. Furthermore, *slx-1*(*tm2644*) mutants, which encode for a catalytically inactive (nuclease negative) protein, show accumulation of recombination intermediates in both mitotic and meiotic cells as well as increased sensitivity to DNA damage inducing agents. These results suggest that SLX-1 is required for DNA repair by processing repair intermediates through its nuclease activity. However, while double Holliday junction resolution is required for crossover formation during meiosis, meiotic crossover frequencies were not reduced in *slx-1*(*tm2644*) mutants, and instead, crossover distribution was altered compared to wild type. Therefore, although SLX-1 is not an essential nuclease for crossover formation via double Holliday junction resolution between homologous chromosomes during meiotic recombination, our studies reveal that SLX-1 plays a role in regulating crossover distribution. This regulation is not mediated through changes in either the levels or distribution of DNA double-strand breaks (DSBs) along chromosomes. Instead, we propose a model in which SLX-1 regulates meiotic crossover distribution such that they occur at the terminal thirds of chromosomes by promoting DSB repair via a noncrossover pathway along the mid-section of chromosomes.

## Results

### SLX-1 cleaves branched DNA in a HIM-18–dependent manner

**Figure 1:**
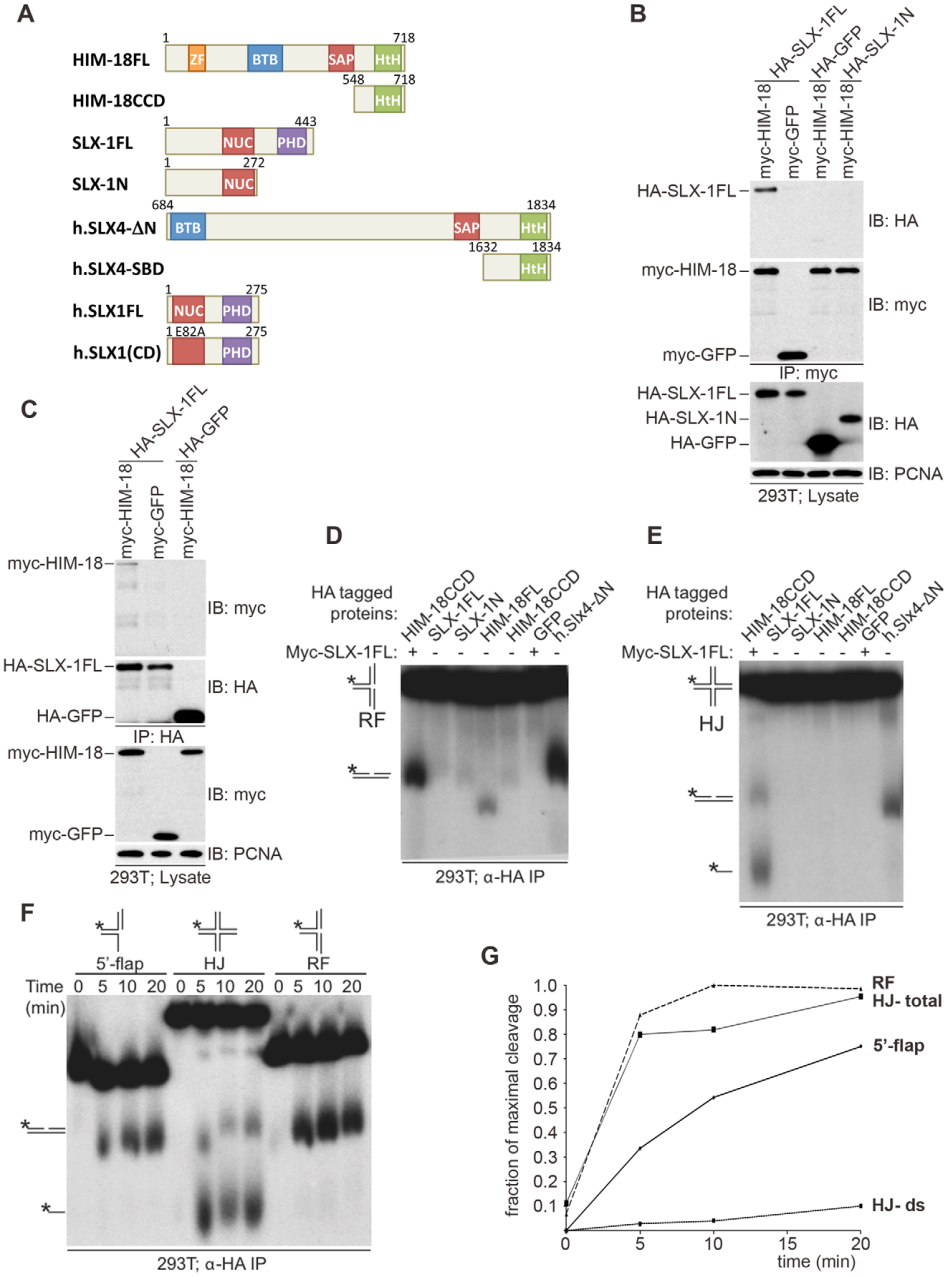
*C. elegans* SLX-1 cleaves branched substrates in a HIM-18–dependent manner. (A) Protein constructs used in this study. Both the length of amino acid residues and domains are shown. FL: full length; CCD: conserved C-terminal domain; SBD: SLX1 binding domain; CD: catalytic dead; ZF: zinc finger; BTB: Broad-Complex, Tramtrack and Bric a brac; SAP: SAF-A/B, Acinus and PIAS; HtH: helix turn helix; NUC: nuclease; PHD: plant homeo domain. (B, C) HIM18 associates with full-length SLX1. (B) The indicated HA- and myc-tagged constructs were transfected into HEK293T cells, the cells lysed and the lysates immuno-precipitated with α-HA resin (Sigma) and immuno-blotted with the indicated antibodies. (C) As in (B), but the lysates were immunoprecipitated with α-myc resin (Sigma). (D–F) Cleavage activity of HIM-18/SLX-1. (D) The indicated proteins were transiently expressed and immuno-purified from 293T cells, incubated with a 32P-end-labeled replication fork (RF) substrate, the products were separated by native gel electrophoresis and visualized by autoradiography. The * indicates the labeled strand. The RF substrate and its major cleavage product are indicated to the left of the gel. (E) Similar to (D), but the Holliday Junction (HJ) was used as the substrate. The HJ substrate and its major cleavage products are indicated to the left of the gel. (F) Time-course to evaluate the cleavage rate of branched DNA substrates by HIM-18/SLX-1. Left panel: The HA-HIM-18CCD/myc-SLX-1 complex was transiently expressed and immuno-purified from 293T cells, incubated with the indicated substrates, the reaction was stopped at the indicated time points and the products separated and visualized as in D and E. (G) Quantification of the autoradiograph on the left using ImageJ software. For HJ, two cleavage rates were calculated: one by taking only the double stranded product into account (HJ-ds), and the other by taking both the double stranded and the single stranded products into account.

We previously showed that *C. elegans* SLX-1 interacts with HIM-18 in a yeast two-hybrid assay [16]. To examine whether these two components directly interact with one another, we transiently transfected HEK-293T cells with epitope-tagged HIM-18 and SLX-1 and performed immunoprecipitation experiments. As shown in Figure 1A–1C, full-length HA-SLX-1 associates with full-length Myc-HIM-18, but not with a control Myc tagged protein (Myc-GFP) under the tested conditions. To further characterize the interaction between SLX-1 and HIM-18, we co-expressed the N-terminal domain of SLX-1 that contains its nuclease domain (HA-SLX-1-N; residues 1–272, Figure 1A) with Myc-HIM-18 in 293T cells. In contrast to what was observed with HA-SLX-1 (full-length), Myc-HIM-18 does not associate with HA-SLX-1-N, consistent with yeast two-hybrid results [3]. We also ascertained that the C-terminal domain of SLX-1 that contains its PHD domain (SLX-1-C; residues 273–443) does not interact with HIM-18 (data not shown). These results suggest that the interaction between HIM-18 and SLX-1 is direct and involves both the N-terminal and C-terminal domains of SLX-1.

To examine the roles of HIM-18 and SLX-1 during recombination, we assessed if the HIM-18/SLX-1 complex displayed endonucleolytic activity towards synthetic DNA substrates. HA-HIM-18-CCD (conserved C-terminal domain, residues 548–718) and Myc-SLX-1 were co-expressed in 293T cells and immuno-purified with an α-HA antibody (the CCD domain of HIM-18 was used since it expressed at a higher level than full-length HIM-18). The HA-HIM-18/Myc-SLX-1 complex was incubated with either a radiolabeled replication fork (RF) or a Holliday Junction (HJ) substrate, and the reaction products were separated by native gel electrophoresis and visualized by autoradiography. HA-HIM-18/Myc-SLX-1 exhibited endonucleolytic activity against both RFs and HJs at a level that was comparable to the human HA-SLX4-ΔN complexes purified from 293T cells (Figure 1D and 1E) [5]. Under similar conditions, neither HIM-18 nor SLX-1 alone displayed appreciable catalytic activity against RFs and HJs (Figure 1D and 1E), indicating that SLX-1 is the catalytically active component of the HIM-18/SLX-1 complex. To further characterize its processing activity, we analyzed the substrate preference of HIM-18/SLX-1 and compared its activity against 5′-flap, HJ, and RF substrates. As shown in Figure 1F–1G, time-course experiments revealed that HA-HIM-18/Myc-SLX-1 preferred the RF substrate to the 5′-flap or HJ substrates under the conditions tested. We also observed that HIM-18/SLX-1 had substantially lower activity against the 3′-flap substrate compared to RFs, 5′-flaps, or HJs (Figure S1).

**Figure 2:**
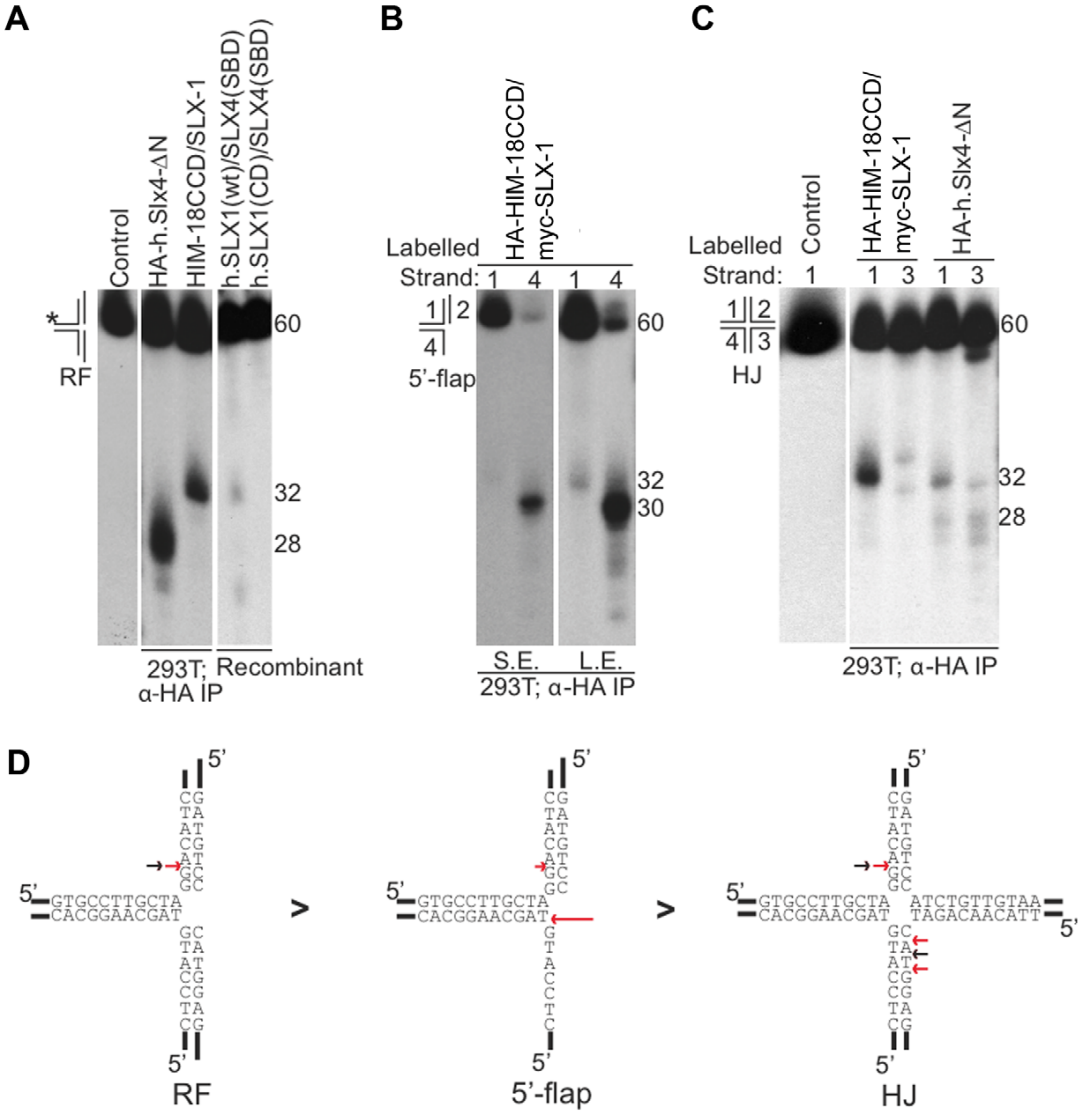
Cleavage specificity of the *C. elegans* HIM-18/SLX-1 complex. (A) Replication fork (RF) substrate was incubated with the indicated proteins complexes transiently expressed and immuno-purified from 293T cells. The reaction products were separated by denaturing gel electrophoresis to determine the site of cleavage. The length of the labeled oligonucleotide reactants or products determined using oligonucleotide markers (not shown) are indicated to the right of the gel. The * indicates the labeled strand. (B) Similar to A, but the substrate used was the 5′-flap. The image on the right was obtained by exposing the gel for a longer time to indicate cleavage on strand 1. S.E., Short exposure; L.E., Long exposure. (C) Similar to A and B, but the substrate used was the Holliday junction (HJ) ^32^P-end-labeled at either strand 1 or 3. In (A)–(C) the source of the protein complexes is indicated below the gel image. (D) Structural model of branched substrates indicating cleavage preference and specificity. The red arrow indicates the cleavage site observed with HIM-18/SLX-1 and the black arrow indicates the major cleavage sites observed with the human SLX4-SBD complex [5]. The length of the red arrow is indicative of the cleavage efficiency.

Next, we determined the cleavage sites of each DNA substrate (Figure 2). We determined that HA-HIM-18/myc-SLX-1 cleaved the RF substrate on strand 1 at 2 nucleotides 3′ to the branch point (Figure 2A and 2D). However, in the case of RFs, the cleavage efficiency against the double stranded region (strand 1) was significantly higher than against the same region of the 5′-flap (Figure 2B). In addition, it should be noted that although human HA-SLX4-ΔN purified from 293T cells had a cleavage specificity against RF that was significantly different to that of HA-HIM-18/myc-SLX-1, recombinant human SLX1/SLX4 (SBD) generated RF cleavage products that included the 32 nucleotide product (two nucleotides 3′ to the branch point) that was also produced by the *C. elegans* protein complex (Figure 2A).

HA-HIM-18/myc-SLX-1 purified from 293T cells cleaved the 5′-flap on strand 3, precisely at the junction between the 5′single-stranded arm and the double stranded region of the substrate similar to 5′-flap cleavage by *Saccharomyces cerevisiae* Slx1-Slx4 [1]. Cleavage also occurred on strand 1 (i.e. the double-stranded region of the 5′-flap), although with much less efficiency than strand 4, at 2 nucleotides 3′ to the branch point (Figure 2B and 2D).

Since HIM-18/SLX-1 displayed significant cleavage activity against HJs (Figure 1D and 1E), we sought to determine if it was in fact a canonical HJ resolvase. A characteristic of bona fide HJ resolvases such as bacterial RuvC, human GEN1 and human SLX1/SLX4, is the ability to cleave opposing strands of a HJ in a symmetric manner to generate products that can be directly ligated [3]–[5], [17], [18]. To test this possibility, we performed cleavage assays against a HJ substrate radiolabeled at either strands 1 or 3 and analyzed the reaction products by denaturing gel electrophoresis. As shown in Figure 2C and 2D, HA-HIM-18/myc-SLX-1 cleaved the HJ substrate at a unique site on strand 1, which was two nucleotides 3′ to the branch point. On strand 3, however, HA-HIM-18/myc-SLX-1 displayed substantially lower cleavage activity compared to strand 1 and cut the HJ at two sites, respectively 1 and 3 nucleotides 3′ to the branch point. This was in contrast to the human HA-SLX4-ΔN complex purified from 293T cells, which cleaves the HJ with perfect symmetry on strands 1 and 3 two nucleotides 3′ to the branch point (Figure 2C and 2D) [5]. These data indicate that the *C. elegans* HIM-18/SLX-1 complex, though displaying cleavage activity against a HJ substrate, does not appear to function as a bona fide HJ resolvase and is reminiscent of *S. cerevisiae* and *Schizosaccharomyces pombe* Slx1-Slx4 endonucleases [1], [2] under the tested conditions.

### *tm2644* encodes for a catalytically inactive (nuclease-negative) SLX-1

**Figure 3:**
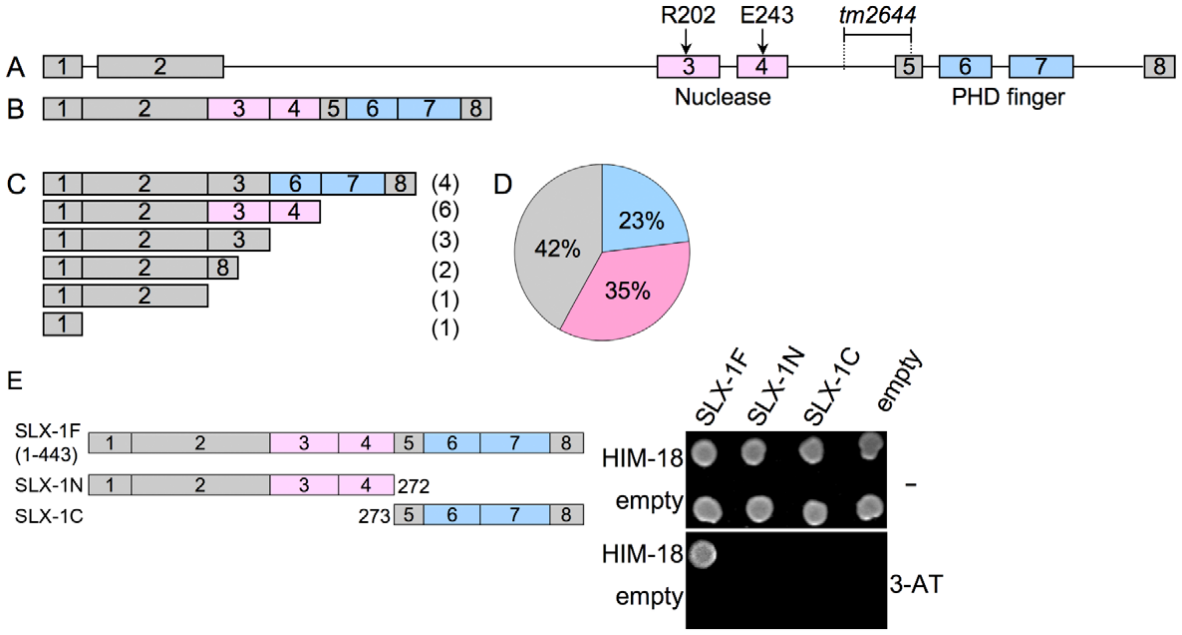
*slx-1*(*tm2644*) mutants express splice variants that lack the C-terminus region required for an interaction with HIM-18, therefore encoding for nuclease-negative variants of SLX-1. (A) Schematic representation of the *slx-1* gene structure. The locations of the nuclease (red) and the PHD finger (blue) domains are shown. R202 and E243 are known catalytic sites of the GIY-YIG nuclease domain. The region deleted in *tm2644* is indicated. (B) *slx-1* cDNA in wild type. (C) *slx-1*(*tm2644*) variant cDNAs. Only start to stop codons are depicted. The number of times each splice variant was detected is indicated in parentheses at the right. (D) The chart indicates the frequency of cDNA variants observed in *slx-1(tm2644)* mutants. Blue, cDNAs including an intact PHD finger domain (exons 6 and 7). Pink, cDNAs including an intact nuclease domain (exons 3 and 4). Gray, cDNAs carrying neither PHD finger nor nuclease domains. (E) Yeast two-hybrid analysis of interactions between full-length HIM-18 fused to GAL4-AD and full-length or truncated SLX-1 fused to GAL4-DB. Interactions were scored by growth on –HIS+1 mM 3-AT plates.

The *slx-1*(*tm2644*) mutant, obtained from the Japanese National Bioresource Project, carries a 205 bp deletion encompassing parts of intron 4 and exon 5 which removes the splice acceptor site of the downstream exon 4 (Figure 3A). RT-PCR using a primer set located between the start and stop codons of the *slx-1* gene revealed that there are several splice variants containing premature stop codons in *slx-1*(*tm2644*) mutants (Figure 3C). Sequence analysis of the RT-PCR products revealed that this alternative splicing does not occur in wild type (Figure 3B). SLX-1 harbors both a GIY-YIG nuclease domain and a PHD finger domain (Figure 3B). 42% of the splice variants lack both the nuclease and PHD finger domains (Figure 3D), whereas 23% contain an intact PHD finger domain (exons 6 and 7) and 35% contain an intact nuclease domain (exons 3 and 4) (Figure 3D). Both exons 3 and 4 carry the conserved catalytic sites of the nuclease (R202 in exon 3 and E243 in exon 4). Moreover, both catalytic sites have been shown to be essential for the nuclease activity of SLX1 both in fission yeast (R34 and E74) [2] and in humans (R41 and E82) [3]. Importantly, the nuclease activity of SLX-1 requires a physical interaction with HIM-18, which is a homolog of human SLX4 (Figure 1D and 1E). However, yeast two-hybrid and immunoprecipitation assays revealed that SLX-1N^1–272^, which is potentially expressed in *slx-1*(*tm2644*) mutants and stems from the only splice variant still carrying the nuclease domain, does not bind to HIM-18 and lacks a nuclease activity (Figure 1A–1E and Figure 3E). Therefore, these results suggest that *slx-1*(*tm2644*) mutants are loss-of-function for SLX-1's nuclease activity.

### SLX-1 shares overlapping roles with the Bloom syndrome helicase and the XPF-1 and GEN-1 structure-specific endonucleases

To investigate whether *slx-1* plays a role in either mitotic development or meiosis, and examine the genetic interactions between *slx-1* and other genes implicated in processing recombination intermediates during these cell division programs, we measured the brood size, embryonic lethality, larval arrest and the incidence of males observed among *slx-1*, *him-6/BLM*, *xpf-1* and *gen-1* mutant offspring (Table 1). A decreased brood size is suggestive of increased sterility, whereas either increased embryonic lethality or larval arrest are suggestive of mitotic defects. Finally, a high incidence of males (Him phenotype) is indicative of increased X chromosome nondisjunction and correlates with meiotic defects, whereas a combination of increased embryonic lethality accompanied by a high incidence of males is suggestive of increased aneuploidy resulting from meiotic missegregation of both autosomes and the X chromosome, respectively [19]. *slx-1* mutants showed a 32% reduction in brood size (P<0.0001), a 4.6-fold increase in embryonic lethality (P = 0.0006), and a 2-fold increase in larval arrest (P = 0.00197) compared to wild type, supporting a role for *slx-1* in mitotic repair. Moreover, a 1.2-fold increase in larval arrest (P = 0.0417) was observed in *slx-1;him-18* double mutants compared to *him-18* single mutants. This marginally lower significance cutoff most likely indicates that SLX-1 is dependent on HIM-18 during larval development. However, we cannot rule out the possibility that SLX-1's function is not completely HIM-18-dependent during this developmental process. Next, we investigated the genetic interaction of SLX-1 with HIM-6, a *C. elegans* homolog of yeast Sgs1 and the human Bloom syndrome helicase, which is required for double Holliday junction dissolution [7]. *slx-1;him-6* double mutants showed synthetic lethality compared to either single mutant (P<0.0001). These results suggest that SLX-1 and HIM-6 function in parallel or alternate pathways, similar to budding yeast [6] and flies [20]. Finally, we examined the genetic interactions between *slx-1* and the structure-specific endonucleases *xpf-1* and *gen-1*, which are a component of the HIM-18 complex and a Holliday junction resolving enzyme, respectively [16], [21]. *slx-1;xpf-1* double mutants showed a 2.4-fold increase (P<0.0001) in embryonic lethality and a 3.6-fold increase in larval arrest (P = 0.0029) compared with *xpf-1* single mutants. These results suggest that SLX-1 and XPF-1 may have overlapping roles during mitotic development. Interestingly, *slx-1;xpf-1;him-18* triple mutants exhibited similar phenotypes to *xpf-1;him-18* double mutants, supporting our observation that the nuclease activity of SLX-1 is dependent on HIM-18 and therefore an *slx-1* mutation causes no obvious plate phenotypes in an *xpf-1;him-18* background. Analysis of *slx-1;gen-1* double mutants revealed a 69% reduction in brood size (P<0.0001) and a 5.5-fold increase in larval arrest (P = 0.0079) compared with *slx-1* single mutants. These results suggest that SLX-1 and GEN-1 share similar, albeit independent, mitotic roles at least during larval development.

**Table 1:** Plate phenotypes.

Genotype	Mean no. of eggs/brood (n)^a^	% Inviable embryos (n)^b^	% Males (n)^c^	% Larval arrest (n)^d^
wild type	312 (10)	1.6 (3129)	0.2 (3026)	1.7 (3079)
*slx-1*	211 (10)	7.3 (2108)	0.4 (1885)	3.5 (1954)
*+/slx-1*	311 (12)	0.3 (3729)	0.1 (3676)	1.2 (3719)
*him-18* e	196 (14)	79.9 (2748)	11.9 (243)	56.1 (553)
*slx-1;him-18*	177 (10)	79.7 (1411)	17.1 (117)	67.4 (359)
*him-6* e	252 (9)	59.1 (2270)	13.7 (878)	5.4 (928)
*slx-1;him-6*	61 (10)	97.7 (611)	ND (1)	92.9 (14)
*xpf-1* e	235 (10)	20.2 (2348)	4.5 (1718)	8.3 (1873)
*slx-1;xpf-1*	159 (13)	49.2 (2069)	3.1 (739)	29.7 (1051)
*xpf-1;him-18* e	120 (29)	84.3 (3842)	13.0 (123)	77.4 (545)
*slx-1;xpf-1;him-18*	99 (16)	85.4 (1578)	13.6 (59)	74.5 (231)
*gen-1*	349 (10)	0.6 (3486)	0.0 (3440)	0.7 (3465)
*slx-1;gen-1*	98 (11)	8.5 (1083)	0.3(800)	19.3 (991)

Parentheses indicate the total number of:

a singled hermaphrodites for which entire brood sizes were scored,

b fertilized eggs scored,

c adults scored,

d L1-L4 worms,

e data from [16].

ND, not determined due to n = 1.

### Recombination intermediates accumulate in *slx-1*(*tm2644*) mutants

**Figure 4:**
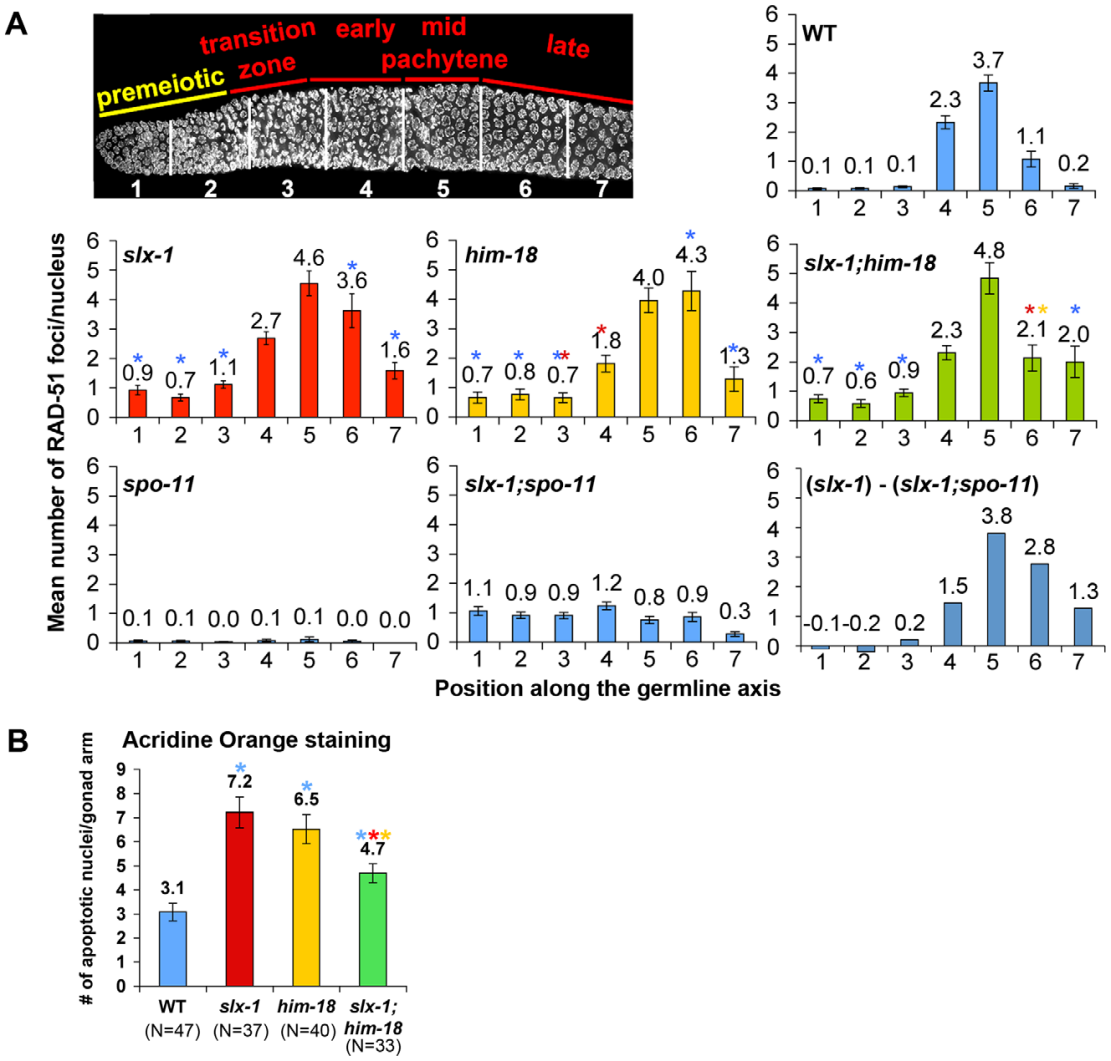
SLX-1 and HIM-18 are required for both mitotic and meiotic DNA repair. (A) Mean number of RAD-51 foci per nucleus. Histograms depict the quantitation of RAD-51foci in germlines of the indicated genotypes. Quantitative analysis of RAD-51 foci depicted in Figure S3, is represented here as the mean number of RAD-51 foci observed per nucleus (y-axis) on each zone along the germline axis (x-axis) for all indicated genotypes. Error bars represent standard error of the mean. To manifest the levels of meiotic RAD-51 foci, mean number of RAD-51 foci in *slx-1;spo-11* double mutants at each zone was subtracted from *slx-1* mutants ((*slx-1*) - (*slx-1;spo-11*)). Colored asterisks indicate statistical significance between different genotypes (wild type, *slx-1, him-18 and slx-1;him-18*). (B) Quantitation of germline apoptosis. Apoptotic corpses stained by acridine orange were scored. N = number of gonad arms scored for each genotype. Colored asterisks indicate statistical significance between different genotypes.

Homologous recombination provides for the repair of both spontaneous DSBs, stemming from stalled or collapsed replication forks at S phase, and programmed DSBs, produced by SPO-11 during prophase of meiosis I. The organization of nuclei in a temporal and spatial gradient in the *C. elegans* germline facilitates the identification and analysis of specific stages of both mitotic and meiotic nuclei. Specifically, nuclei at the distal tip end are undergoing mitotic proliferation (zones 1 and 2), nuclei at the transition zone are in the leptotene/zygotene stages of meiosis (zone 3), followed by nuclei in early pachytene (zone 4), mid pachytene (zone 5) and late pachytene (zones 6 and 7) (Figure 4A). To investigate whether SLX-1 is required for the maintenance of genomic integrity in the *C. elegans* germline, we monitored the levels as well as the kinetics of appearance and disappearance of RAD-51, a protein involved in strand invasion/exchange during DSB repair (Sung, 1994; Colaiacovo et al. 2003). Quantification of RAD-51 foci revealed that these were elevated in both mitotic (zones 1 and 2) and meiotic (zones 3, 6 and 7) nuclei in *slx-1* mutants compared to wild type (Figure 4A and Figure S2). Moreover, the mitotic RAD-51 foci persist through late meiotic prophase (late pachytene stage; zone 6) as observed in *slx-1;spo-11* double mutants which lack the formation of programmed meiotic DSBs (Figure 4A and Figure S2). However, simple subtraction of the number of RAD-51 foci in *slx-1; spo-11* from the number observed in *slx-1*, as a means of approximating the dynamics of SPO-11-dependent DSBs, also reveals an increase of meiotic recombination intermediates during late pachytene in *slx-1* mutants. Further support for a role for SLX-1 in germline DNA repair stems from our analysis of germ cell apoptosis, which was increased 2.3-fold in *slx-1* mutants compared to wild type (Figure 4B). Increased germ cell apoptosis was previously shown to occur when an inability to repair DNA damage results in the activation of a DNA damage checkpoint in late pachytene [22]. Taken together, these results suggest that SLX-1 is required for the proper repair of both stalled/collapsed replication forks and meiotic DSBs.

### SLX-1 is required for replication-coupled DNA repair

**Figure 5:**
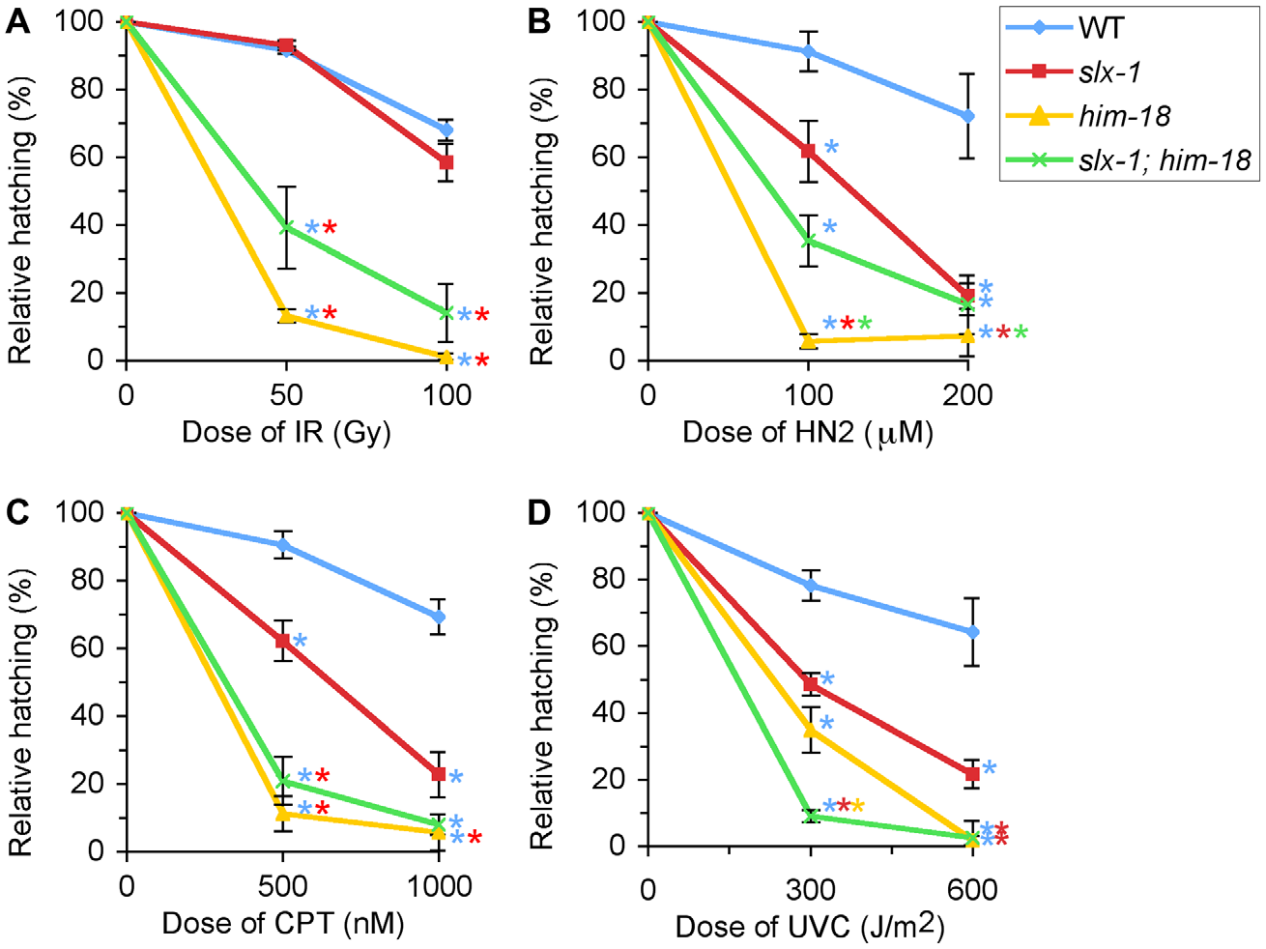
*slx-1* mutants exhibit hypersensitivity to nitrogen mustard, camptothecin, and ultraviolet C, but not ionizing radiation. Relative hatching frequencies detected in wild type, *slx-1, him-18* and *slx-1;him-18* mutants after treatment with the indicated doses of (A) ionizing radiation (IR), (B) nitrogen mustard (HN2), (C) camptothecin (CPT), and (D) ultraviolet C (UVC). Hatching is plotted as a fraction of the hatching observed in untreated animals. Error bars indicate standard error of the mean for ∼20 animals in each of three independent experiments. Colored asterisks indicate statistical significance between different genotypes.

To further investigate which kinds of DNA damage are repaired by SLX-1-HIM-18 *in vivo*, we performed a series of DNA damage sensitivity assays by exposing *slx-1* mutants to γ-irradiation, which produces DSBs, nitrogen mustard (HN2), which produces DNA inter-strand crosslinks, camptothecin (CPT), which results in single-strand nicks, and UVC, which causes cyclobutane pyrimidine dimers (Figure 5).

After treatment with g-irradiation, no statistically significant, reduction in hatching ratio was observed in *slx-1* mutants compared to wild type (P = 0.5512 and P = 0.1455, respectively at 50 and 100 Gy) (Figure 5A). In contrast, hatching ratios were significantly reduced in *him-18* (P<0.0001 and P<0.0001) and *slx-1;him-18* (P = 0.0004 and P = 0.0002) double mutants after doses of either 50 or 100 Gy of irradiation (Figure 5A). These results suggest that HIM-18 plays a role in the repair of exogenously induced DSBs, which is independent of the nuclease activity of SLX-1.

Exposure to HN2 revealed that *slx-1* and *slx-1;him-18* mutants share similar hypersensitivity to HN2 compared to wild type at both 100 µM and 200 µM, while *him-18* mutants showed more severe hypersensitivity compared to *slx-1* and *slx-1;him-18* mutants (Figure 5B). These results could be explained by the fact that SLX-1 function is HIM-18-dependent. Therefore, in *him-18* mutants ICL repair may be further affected by the presence of inactive SLX-1.

Exposure to CPT revealed hypersensitivity among *slx-1* mutants compared to wild type at both 500 nM and 1000 nM doses (Figure 5C). Moreover, *him-18* and *slx-1;him-18* mutants showed more severe hypersensitivity compared to *slx-1* mutants. Since CPT inhibits the removal of topoisomerase I, thereby forming nicked sites after replication [23], these results suggest that SLX-1 is either required for efficient removal of the TOP1-CPT complex or resolution of a HJ intermediate during the re-establishment of a replication fork. Extrapolating from observations made in other species [1], [5], loss of HIM-18 may reduce the nuclease activity of SLX-1, MUS-81 and potentially XPF-1. This would explain why *him-18* and *slx-1;him-18* mutants show similar hypersensitivity to CPT.

Notably, *slx-1*, *him-18* and *slx-1;him-18* mutants showed hypersensitivity to UV (Figure 5D), although the loss of either SLX1 or SLX4 orthologs does not result in hypersensitivity in budding yeast [8], fission yeast [2], flies [24], mouse [13] and human cells [3], [5]. Whereas mutants in several other DNA repair genes such as *mus81* and *sgs1* in *S. cerevisiae* [6], [25] have exhibited UV sensitivity, but have no proven direct role in nucleotide excision repair (NER), one can not discard the possibility that in *C. elegans*, unlike in other species, SLX-1 and HIM-18 may be required for NER.

### SLX-1 does not regulate either the levels or the distribution of meiotic DSBs

**Figure 6:**
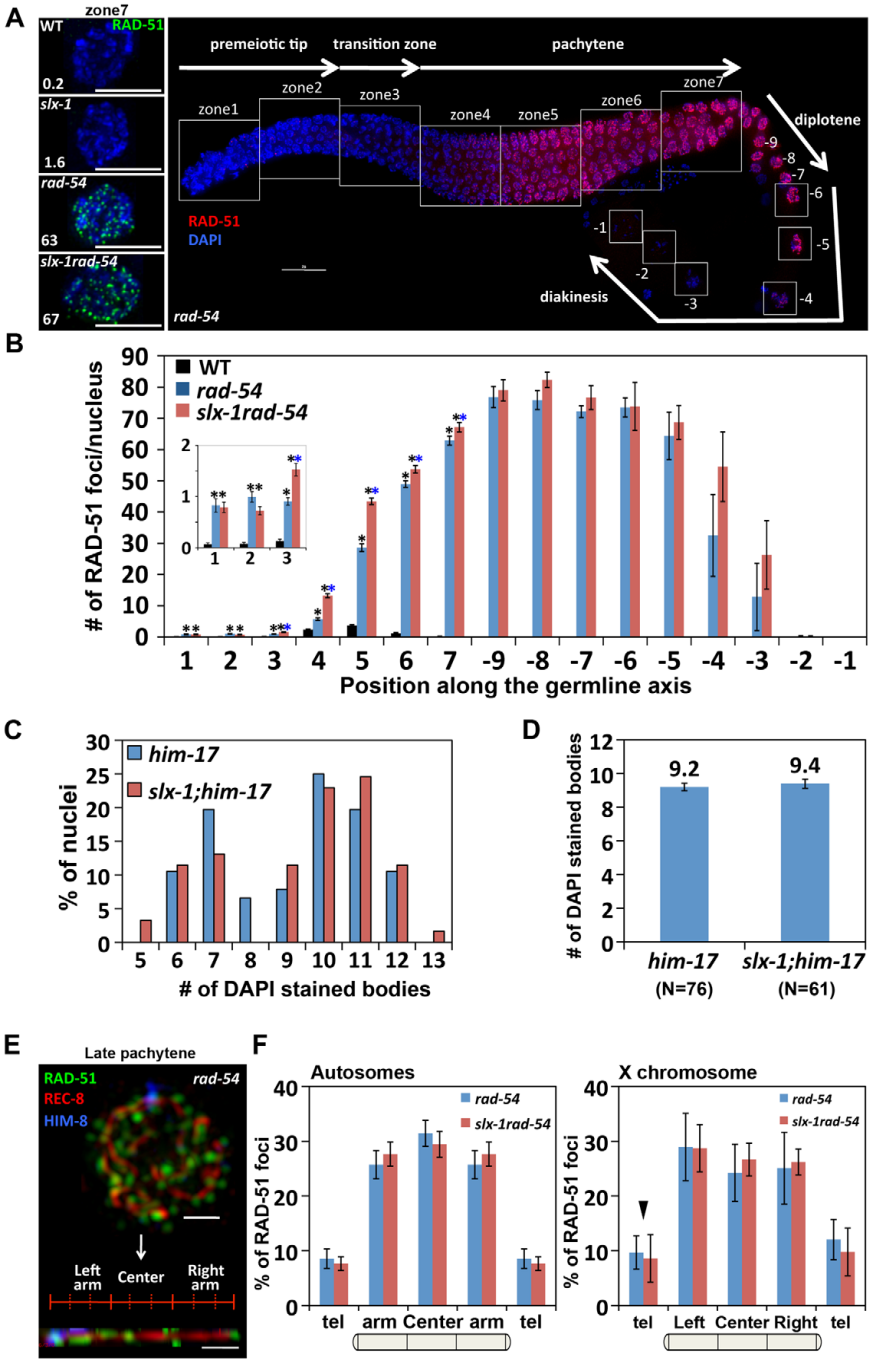
SLX-1 does not regulate either the levels or the distribution of DSBs. (A) Panels on the left are high magnification images of late pachytene nuclei (zone 7) immunostained with RAD-51 in the indicated genotypes. The average number of RAD-51 foci is shown at the bottom left. Bars, 5 µm. The panel on the right shows a typical immunostaining pattern for RAD-51 in whole mounted gonads of *rad-54* mutants. The white squares indicate the seven zones and the diakinesis oocytes (−9 through −1) scored in the analysis. (B) Quantitation of the number of RAD-51 foci in wild type, *rad-54* and *slx-1rad-54* mutants. Error bars indicate standard error. Colored asterisks indicate statistical significance between different genotypes. (C) Quantitation of DAPI-stained bodies at late diakinesis (−1 and −2 oocytes) in six time outcrossed *him-17(ok424)* and *slx-1;him-17(ok424)* mutants. (D) Average number of DAPI-stained bodies in *him-17(ok424)* and *slx-1;him-17(ok424)* mutants. Bars indicate standard error. (E) A representative image of a late pachytene nucleus (zone 7) co-immunostained with RAD-51, REC-8 and HIM-8 antibodies and a computationally straightened chromosome axis (bottom) in *rad-54* mutants. (F) Distribution of RAD-51 foci along chromosome axes in *rad-54* and *slx-1rad-54* mutants. RAD-51 foci localization on randomly selected autosomes are quantified in the left panel. The values of RAD-51 distribution on the left and the right arms are averaged as “arm” on the autosomes. The values at the left and the right telomeres are also averaged as “tel” on the autosomes. The distribution of RAD-51 foci on the X chromosome is shown in the panel at the right. Arrowhead indicates pairing center where HIM-8 localizes. Bars indicate standard error.

To investigate whether the higher levels of RAD-51 foci are due to either an increased number of DSBs or a delay of the repair process in *slx-1* mutants, we quantified DSB levels by RAD-51 immunostaining in *rad-54* mutants, in which DSB repair is blocked and DSB-bound RAD-51 is proposed to be trapped in the germline [26]. In wild type, RAD-51 foci start to increase in nuclei at the entrance into meiosis (zone 3), peak at 3.7 foci/nucleus at mid pachytene (zone 5) and are practically all gone by late pachytene (zone 7) (Figure 4A, Figure 6B and Table S2). In contrast, in *rad-54* mutants, higher levels of mitotic RAD-51 foci were observed (0.8 and 1.0 compared to 0.1 at zones 1 and 2, P<0.0001, respectively), and meiotic RAD-51 foci peaked at 75 foci/nucleus at diplotene (−7 oocyte), only being completely absent by late-diakinesis (−1 oocyte) (Figure 6A–6B and Table S2). These three observations: 1) elevated mitotic RAD-51 foci; 2) peak of RAD-51 foci at diplotene; 3) RAD-51 foci only being completely lost by late diakinesis, are different from those described in [26]. They concluded that RAD-51 foci peak at 12/nucleus at early, mid and late pachytene stages in *rad-54* mutants. However, both our studies as well as those of others have since revealed a higher number of RAD-51 foci in this mutant background (18–30 and 26–63 foci at mid and late pachytene, respectively [27], [28], this current study). Further support for the number of DSBs we observed in the *rad-54* background stems from our analysis of the level of RPA-1-YFP foci in *brc-2* mutants, in which DSB repair is blocked and replacement of RPA-1 (replication protein A) by RAD-51 at the resected DSB ends is inhibited in the germline [29] (Figure S4). We observed a similar number of RPA-1-YFP foci (59.1) in *brc-2* mutants to that of RAD-51 foci (62.9) in *rad-54* mutants in late pachytene nuclei (zone 7). Importantly, we confirmed that the elevated levels of RAD-51 foci we observe in *rad-54* mutants at mid and late pachytene, where the events of repair we are focused on take place, are not already at a possible maximum thus obscuring our ability to utilize this mutant background to identify further increases in DSB levels. Specifically, following the formation of additional DSBs by γ-irradiation in *rad-54* mutants, we observed 45 foci at 50 Gy compared to 29 foci at 0 Gy in zone 5 (P<0.0001) (Figure S3). *slx-1rad-54* double mutants exhibited levels of RAD-51 foci similar to those observed in *rad-54* mutants, although RAD-51 foci levels accumulated with slightly faster kinetics than in *rad-54* mutants. Furthermore, it is known that elevated levels of meiotic DSBs rescue *him-17* mutants, which are deficient in meiotic DSB formation [30], [31]. *slx-1;him-17* double mutants did not rescue the *him-17* mutant phenotype (Figure 6C–6D). Taken together, these results suggest that the total levels of DSBs are wild type in *slx-1* mutants.

In *C. elegans*, as in many other species, crossover formation is biased towards the terminal thirds of autosomes [32], [33]. To measure the levels and distribution of DSBs along chromosomes, and determine whether they are altered in *slx-1* mutants, we performed three-dimensional traces of chromosomes in late pachytene nuclei by visualizing chromosome axes with an antibody to the meiotic cohesin REC-8 and quantified the levels and distribution of recombination intermediates along these chromosome axes with a RAD-51 antibody, comparing *rad-54* and *slx-1 rad-54* double mutants (Figure 6E–6F). To distinguish the X chromosome from the autosomes, we identified the X chromosome pairing center end with an anti-HIM-8 antibody [34]. We did not detect a biased distribution of RAD-51 foci along either the arms or the central region of the chromosomes in *rad-54* mutants. Therefore, this even distribution of DSBs along the lengths of the chromosomes suggests the existence of mechanisms that inhibit crossover formation after the induction of DSBs at the central region of the chromosomes. Interestingly, both the levels and distribution of RAD-51 foci along chromosome axes were similar between *rad-54* and *slx-1 rad-54* mutants in both autosomes and the X chromosomes (Figure 6F). These results suggest that SLX-1 does not alter either the overall levels or the distribution of DSBs along either the X chromosomes or the autosomes.

### SLX-1 is required for the normal crossover landscape

**Figure 7:**
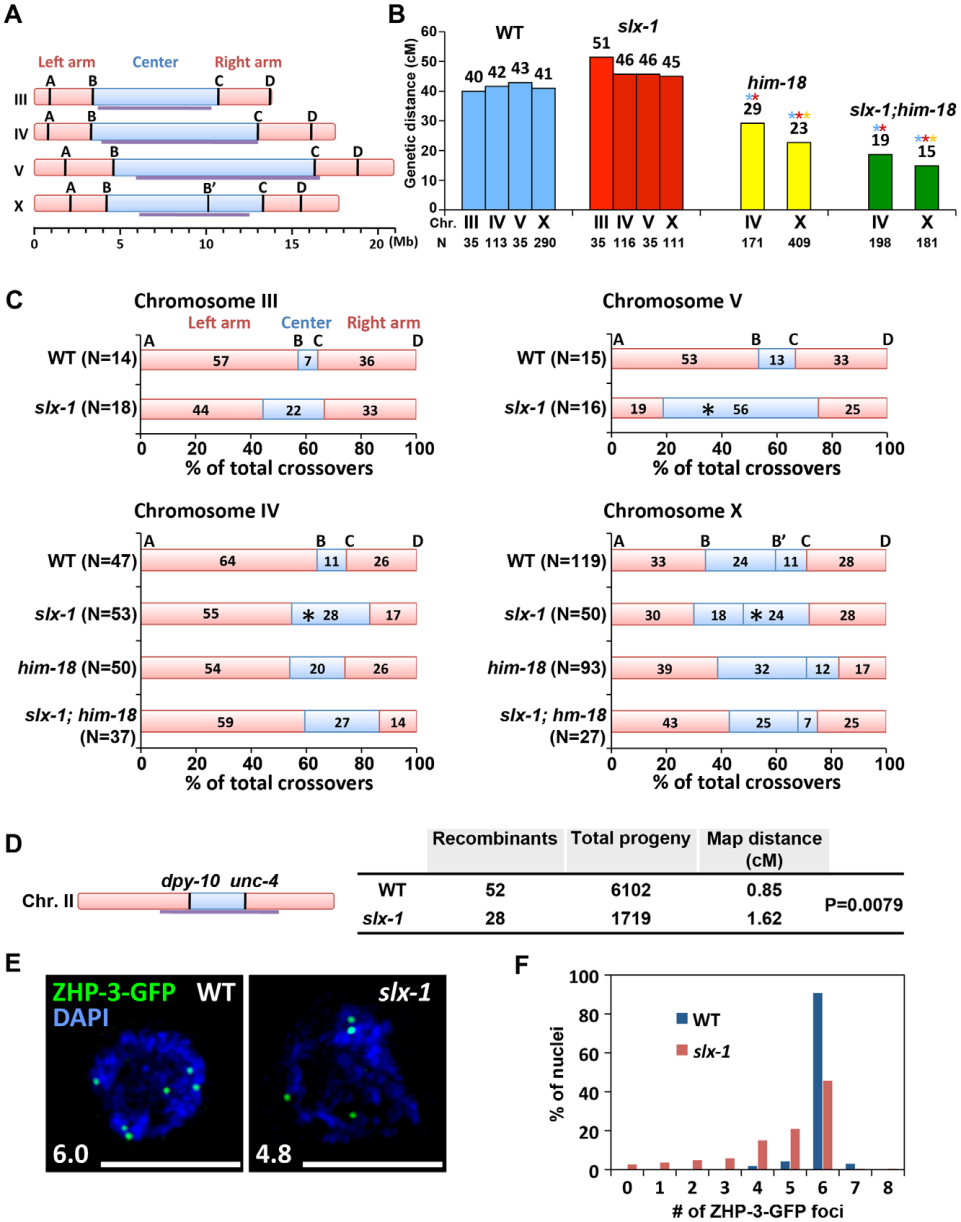
SLX-1 promotes the regulation of crossover distribution but not crossover frequency. (A) Schematic representation of chromosome domains. Four SNP markers for chromosome III, IV and V and five SNP markers for the X chromosome are shown as A, B, B', C and D. The intervals A–B, B–C and C–D indicate portions of the left arm, center, and right arm, respectively. Purple lines indicate the central domain on which crossover frequency is lower compared to the arms defined by Rockman and Kruglyak [79]. (B) Analysis of crossover frequencies on chromosomes III, IV, V and X in wild type, *slx-1*, *him-18* and *slx-1;him-18* mutants. Crossover frequencies are measured between SNP positions A to D. N = number of crossprogeny scored. (C) Analysis of crossover distribution on chromosomes III, IV, V and X on the indicated genotypes. The positions on the chromosomes indicated as left, center and right correspond to intervals A–B, B–C and C–D, respectively as indicated on panel A. Asterisks indicate significant differences compared to wild type. (D) Crossover frequency of wild type and *slx-1* mutants between *dpy-10* and *unc-4* loci on center region of chromosome III. (E) Representative images of ZHP-3-GFP foci in late pachytene nuclei (zone 7) in wild type and *slx-1* mutants. The average number of ZHP-3-GFP foci is shown at the bottom left. Bars, 5 µm. (F) Quantitation of ZHP-3-GFP foci in late pachytene nuclei in wild type and *slx-1* mutants.

To investigate whether SLX-1 and HIM-18 are required for meiotic crossover formation, we first observed crossover frequencies in *slx-1*, *him-18* and *slx-1;him-18* mutants on both chromosomes IV and X by using the snip-SNP method [16], [35]. Crossover frequencies were not significantly different between wild type and *slx-1* mutants on either chromosome (Figure 7A and 7B). However, reduced crossover frequencies were detected in *slx-1;him-18* double mutants compared to *him-18* single mutants. These data coincide with the observation of a lack of a Him phenotype among *slx-1* mutants, whereas *slx-1;him-18* mutants show a more severe Him phenotype compared to *him-18* single mutants (Table 1). Taken together, these results suggest that SLX-1 is not required to make interhomolog crossovers in normal meiosis, but is partially required on both autosomes and X chromosomes in a *him-18* background.

Meiotic DSBs, with the exception of the subset designated to be repaired as future interhomolog crossovers, are repaired either by interhomolog noncrossover or intersister pathways [36], [37]. We examined intersister repair by monitoring chromosome morphology in diakinesis oocytes of *syp-2* and *rec-8* mutants, where either interhomolog interactions or sister chromatid cohesion are impaired, respectively [38]–[40]. We did not observe any additive cytological defects in both *slx-1;syp-2* and *slx-1;rec-8* double mutants compared with *syp-2* or *rec-8* single mutants (Figure S5). While these results suggest that SLX-1 may not function during intersister repair, confirmation awaits additional analysis of sister chromatid separation at anaphase where either lagging chromosomes or chromosome bridges might be detected if there are defects in the intersister resolution of dHJs.

We next used the snip-SNP method to assess crossover distribution along chromosomes III, IV, V and X. In *slx-1* mutants, the frequency of crossovers detected in the center (intervals B–C) of the autosomes (III, IV and V) is higher (3.1-, 4.2- and 2.7-fold increases, respectively) than in wild type, whereas it is reduced in the arm regions (intervals A–B and C–D) (Figure 7C and Table S3). Using a pair of morphological markers we further confirmed the occurrence of a higher crossover frequency at the central region of chromosome II in *slx-1* mutants compared to wild type (Figure 7D). Crossover distribution along the X chromosome is also different between wild type and *slx-1* mutants. At the right portion of the central region of the X chromosome (B'–C), the frequency of crossovers in *slx-1* is higher (2.2-fold increase, P = 0.0348) than in wild type. These results suggest that SLX-1 is required for proper crossover distribution along both autosomes and the X chromosome. To further examine the role of SLX-1 in crossover distribution regulation, we measured crossover distribution on chromosomes IV and X in *him-18* and *slx-1;him-18* double mutants. Crossover levels are increased at the center of chromosome IV in both *him-18* and *slx-1;him-18* double mutants compared with wild type (1.9- and 2.5-fold increases, respectively). Moreover, while the observed increases are similar between *slx-1* and *slx-1;him-18*, only a moderate increase is detected in *him-18* mutants on chromosome IV (Figure 7C and Table S3). Given that SLX-1 is nearly catalytically dead with regard to its nuclease activity in the absence of HIM-18 *in vitro* (Figure 1D–1E), the nuclease activity of SLX-1 may not work in *him-18* mutants *in vivo*. Instead, it is possible that other functions of SLX-1, for example the PHD finger-dependent recognition of epigenetic marks, might still work in *him-18* mutants. Taken together, these results suggest that the nuclease activity of SLX-1 may be important to maintain proper crossover distribution.

It has been proposed that only one of the various DSB sites along a chromosome is specifically designated as a future interhomolog crossover site [41]. ZHP-3, a homolog of *S. cerevisiae* Zip3 protein, has been proposed to mark crossover precursor sites during late pachytene stage in *C. elegans* [16], [42]. To investigate whether crossover designation properly occurs in *slx-1* mutants, we compared the numbers of ZHP-3-GFP foci present in pachytene nuclei in wild type and *slx-1* mutants. The average number of ZHP-3-GFP foci observed in *slx-1* mutants is 80% of those in wild type (Figure 7E). Specifically, while nearly 90% of late pachytene nuclei contain six ZHP-3 foci in wild type, only 45% of nuclei contain six and 55% have less than five ZHP-3-GFP foci in *slx-1* mutants (Figure 7F). Given that crossover levels are indistinguishable between wild type and *slx-1* mutants, these results suggest that a crossover pathway that is not associated with ZHP-3 foci exists in *slx-1* mutants. Alternatively, it could be possible that SLX-1 is required for proper crossover designation.

## Discussion

### SLX-1 has a structure-specific endonuclease activity

In budding yeast, the substrate preference observed for recombinant Slx1-Slx4 is 5′-flaps>Y forks>RFs>mobile HJs>3′-flaps>fixed HJs (the latter involves an asymmetric cut given the non-ligatable processed substrate that is then detected) (Flott and Brill 2003). In fission yeast, the Slx1 immunoprecipitation product cuts stem loops and HJs (symmetric cut) [2]. Finally, in humans, SLX1/SLX4 exhibits preference for 5′flaps and HJs>RFs>3′-flaps [3], [5].

In this current study, we determined that in *C. elegans*, the preference of SLX-1-HIM-18 is for RFs>5′-flaps>HJs>3′-flaps (Figure 1F–1G and Figure 2D). The slight difference observed in the order of substrate preference in *C. elegans* compared to those in yeast and humans is thought to originate from either the difference of the growth temperature or the difference of the length of the N-terminal domain of SLX-1 (168 amino acids compared to 9 amino acids in both the yeast and human orthologs) (Figure 1A). Future analysis may require performing the *in vitro* nuclease assay at 20°C, which is the optimum temperature for growth of *C. elegans*, and using an N-terminal truncated SLX-1. Similar to other organisms, the nuclease activity of SLX-1 depends on HIM-18 (Figure 1D–1E). This endonuclease activity potentially affects the homologous recombination machinery during ICL-repair, break-induced replication and NER. During prophase of meiosis I, homologous recombination occurs between homologous chromosomes. Double Holliday junction resolution is important for crossover formation, however the HJ resolvase activity of the SLX-1-HIM-18 complex is not required for meiotic crossover formation (Figure 7B). Potentially, other structure-specific endonucleases such as XPF-1, which interacts with HIM-18 [16], MUS-81 and GEN-1 [21], may act coordinately to resolve dHJs during meiotic recombination.

### The mitotic roles of SLX-1 in various DNA damage repair pathways

**Figure 8:**
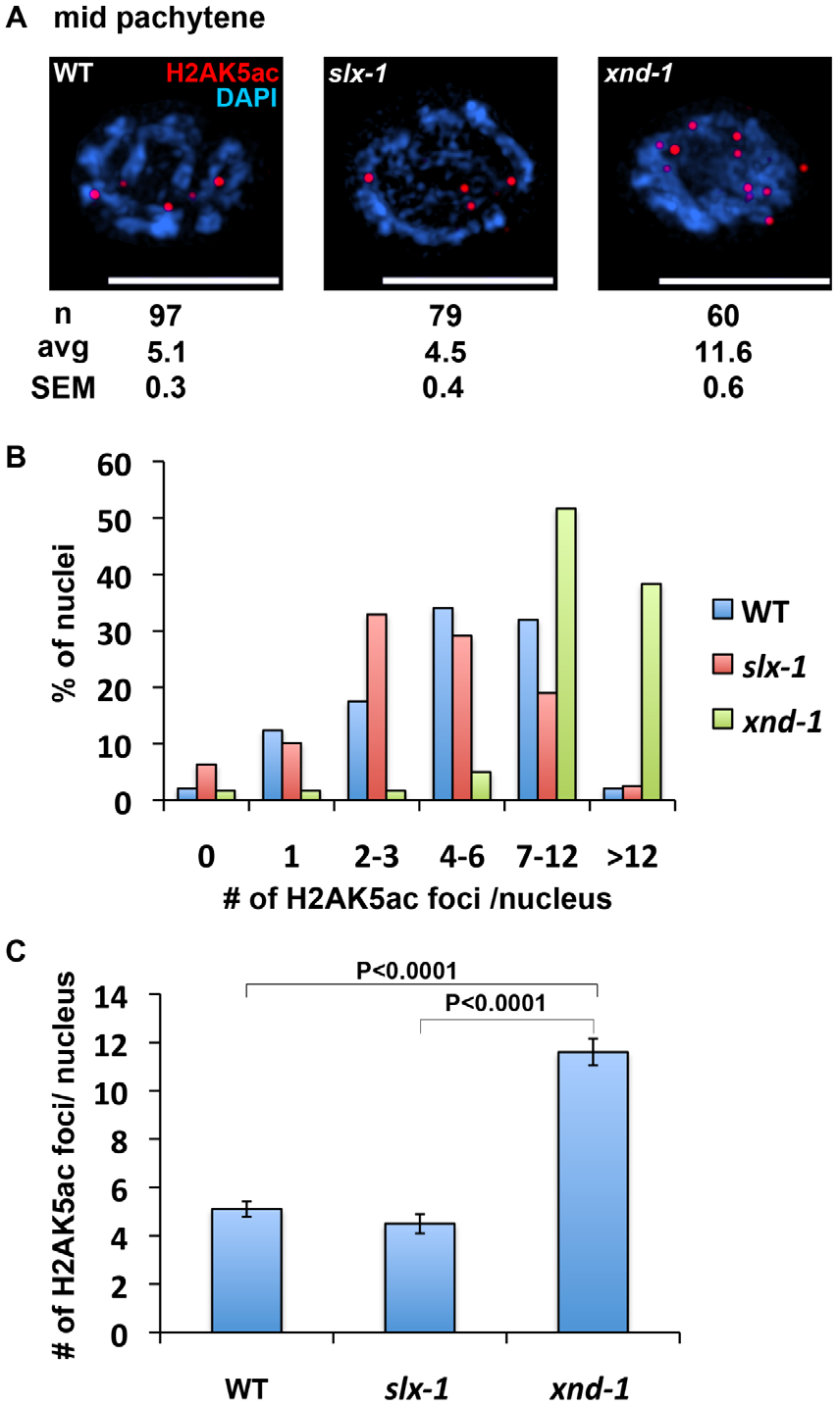
The levels of H2AK5ac are similar between wild type and *slx-1* mutants. (A) Representative images of mid-pachytene nuclei stained with anti H2AK5ac in wild type, *slx-1* and *xnd-1* mutants. Bars, 5 µm. (B) Quantitation of H2AK5ac foci at mid pachytene in wild type, *slx-1* and *xnd-1* mutants. (C) Average number of H2AK5ac foci/nucleus in wild type, *slx-1* and *xnd-1* mutants. Bars indicate standard error.

We showed that *slx-1* mutants were hypersensitive to several kinds of DNA damaging agents (Figure 4C and Figure 8A). Notably, both *slx-1* and *him-18* mutants showed hypersensitivity to UV. This phenotype is different from that observed in budding yeast, fission yeast, flies, mice and humans [2], [3], [5], [8], [13], [24]. SLX-1 has a GIY-YIG nuclease domain also present in UvrC in *E. coli* where it is important for making an incision 3′ to the damage site (cyclobutane pyrimidine dimer, CPD) during the NER process [43], [44]. Additionally, the N-terminal domain of *C. elegans* SLX-1 is longer than that of its homologs in other organisms, so it is possible that the N-terminal region confers the NER function of SLX-1. XPF is largely known as a repair factor for the NER pathway, including in *C. elegans* [45], [46]. XPF exists in two types of complexes in human cells, one is a 2M Dalton complex containing SLX1, SLX4, MUS81, EME1 and ERCC1, the other is the XPF-ERCC1 heterodimer [4]. Therefore, it remains to be determined whether XPF-1, SLX-1 and HIM-18 make single or heterologous complexes during different DNA damage responses or at different stages of the cell cycle.

It is thought that the dual incisions surrounding an interstrand crosslink are performed by MUS81, XPF or FAN1 [47]–[52]. In this study, we raise the possibility that SLX-1 might have the activity required for the incision based on the HN2 hypersensitivity observed in *slx-1* mutants (Figure 5B and Figure 8A). Surprisingly, the mutation in *slx-1* partially suppressed the HN2 induced DNA damage sensitivity observed in *him-18* mutants (Figure 5B). It has been reported that SLX1 represses the nuclease activity towards RFs and 3′-flaps of MUS81 and XPF in human cells [4]. Therefore, in the *him-18* mutant, SLX-1 might repress the incision activity of MUS81 and XPF or other nucleases such as FAN1. Given that HIM-18 carries sites potentially recognized by kinases and ubiquitin/SUMO conjugating enzymes [16], that both human and yeast Slx4 are phosphorylated by ATM/ATR [53], and that human SLX4 is phosphorylated by PLK1 [5], it is possible that post-translational modifications of HIM-18 might modulate its ability to regulate the nuclease activity of the components of the HIM-18 complex either directly or indirectly. In addition to HN2 hypersensitivity, both accumulation of RAD-51 foci in late pachytene (zone 6) and germ cell apoptosis in *him-18* mutants are partially rescued by the *slx-1* mutation (Figure 4A and 4B). One possible explanation is that SLX-1 might be deregulated in the absence of HIM-18 in these cases. In *him-18* mutants, inactive SLX-1 might inhibit these repair pathways. In *slx-1;him-18* double mutants, since there is no inhibition by SLX-1, the *him-18* phenotype is partially suppressed. Further studies will reveal the regulation of the HIM-18 complex in each DNA repair pathway.

### Catalytic subunits of a HJ resolvase for meiotic crossover formation

We showed that SLX-1 is not required for wild type levels of crossover formation during meiotic recombination (Figure 7B). It is believed that there are two possible pathways that can lead to crossover formation, one is double Holliday junction resolution [54] and the other is a “nick/counternick” mechanism [55]. A possible explanation for why crossover frequency is not changed in *slx-1* mutants is that SLX-1 and other structure specific nucleases, such as GEN-1, MUS-81 and XPF-1, are partially redundant and can compensate for each other with regards to the activity of crossover formation. This is supported in part by the observations that *gen-1* mutants are fertile [21] (Table 1), *mus-81* mutants do not enhance X chromosome non-disjunction [16] and *xpf-1* mutants exhibit only a mild reduction in crossover levels [16]. Moreover, *slx-1* enhances the developmental defects observed in *xpf-1* and *gen-1* mutants, supporting the idea that functions of SLX-1 are partially redundant with those of GEN-1 and XPF-1 (Table 1). Furthermore, a recent study suggests that MUS81, SLX4 and GEN1 can compensate for lack of the BLM helicase in human cells by resolving HJs in somatic Bloom's syndrome cells [56]. Therefore, further investigation of the genetic interactions between these structure-specific endonucleases may reveal whether there are redundancies for the activities of Holliday junction resolution during meiotic recombination. Another aspect to consider is that excess crossovers generated in wild type following X-ray exposure are dependent on MUS-81 in *C. elegans*, although MUS-81 is not required for physiological crossover formation during meiosis [57]. *mus-81* and *slx-1* mutants share a couple of phenotypes that are not observed in either *xpf-1* or *gen-1* mutants, such as the elevated levels of RAD-51 foci observed during mitotic proliferation (Figure 4A and Figure S2) [16] and the synthetic lethality with *him-6* (Table 1) (Saito et al., unpublished results). Therefore, it remains to be determined whether SLX-1 is also required for crossover formation under an excess of DSBs resulting from IR treatment in a manner similar to *mus-81* mutants.

### SLX-1 might function as either a noncrossover-specific resolvase of double Holliday junctions or an epigenetic reader during meiosis

How is crossover distribution regulated in *C. elegans* meiosis? Recently, it was reported that crossover distribution is shifted from the arms to the center of chromosomes in *xnd-1* mutants, a phenotype reminiscent to that we observed in our current analysis of *slx-1* mutants [58]. Hyperacetylation of histone H2A lysine 5 is one of the features of the *xnd-1* mutants. However, the acetylation levels of H2AK5 are similar to those of wild type in *slx-1* mutants (Figure 7). Therefore, the hyperacetylation of H2AK5 is not the cause of the change of crossover distribution in *slx-1* mutants.

**Figure 9:**
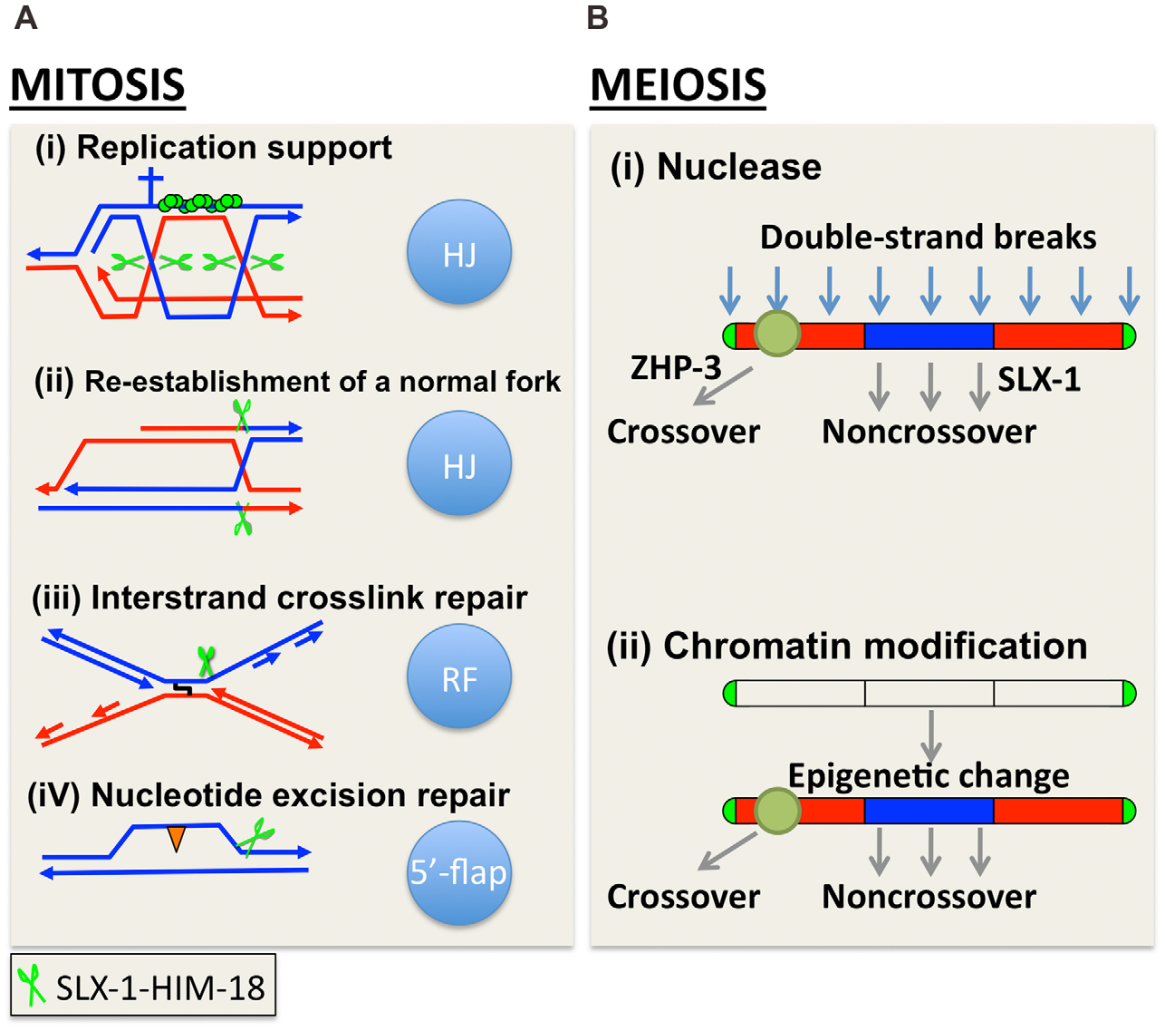
A model for how SLX-1 functions as a structure-specific endonuclease in various genome maintenance pathways. (A) Mitotic role of SLX-1. DNA repair pathways and the nuclease activity of SLX-1 are indicated. The HIM-18-dependent nuclease activity of SLX-1 is required for replication-coupled DNA repair such as during the re-establishment of a normal fork, DNA interstrand crosslink (ICL) repair, and nucleotide excision repair (NER). (A-i) The SLX-1-HIM-18 complex supports the RAD-51-mediated (green circles) repair pathway employed at replication forks. Specifically, SLX-1-HIM-18 resolves Holliday junctions when replication forks stall or collapse during S phase. Green scissors represent the SLX-1-HIM-18 nuclease complex. The cross represents a replication block. (A-ii) HIM-18-SLX-1 resolves HJs during re-establishment of a normal replication fork when CPT-induced single-ended DSBs are generated. (A-iii) The SLX-1-HIM-18 complex induces an incision at DNA ICLs when replication forks collide at an ICL. (A-iv) SLX-1-HIM-18 repairs UV-induced CPDs (orange triangles) via the replication coupled repair and NER pathways. Although it is thought that XPF and XPG make the 5′ and 3′ incisions, respectively, flanking the CPD lesion during the NER pathway in other organisms, there may be a possibility that SLX-1 also makes the 3′ incision, which is reminiscent of its 5′-flap cleavage activity. (B) SLX-1 is required for proper crossover distribution during meiotic recombination. Two alternative models are presented. (B-i) SLX-1 is a noncrossover specific Holliday junction resolvase. One of the evenly distributed DSBs are selected as a crossover precursor at one of the arm regions and marked by ZHP-3. DSBs located at the center of the chromosomes are repaired as noncrossovers via double Holliday junction resolution, synthesis-dependent strand annealing (SDSA) or intersister repair. SLX-1 might convert a dHJ into a noncrossover product by same sense dHJ resolution at the mid-section of chromosomes. (B-ii) Although the function of the PHD finger of SLX-1 is unknown, it is possible that a SLX-1-dependent modification or recognition of chromosome domains contributes to the regulation of crossover distribution.

Based on the analysis of both DSB and crossover distribution in *slx-1* mutants, we propose a model in which SLX-1 inhibits crossover formation at the center of the chromosomes during meiotic recombination (Figure 9B). While crossover formation is biased to the arms in wild type, surprisingly we found that DSBs are more evenly distributed along chromosome axes in wild type. Only one of the DSBs, presumably the one marked by ZHP-3, is converted into an interhomolog crossover at one of the arm regions. All other DSBs are repaired either by intersister repair or interhomolog noncrossover formation. Interestingly, the number of ZHP-3 foci is reduced to 80% of wild type levels in *slx-1* mutants, and nevertheless the total number of crossovers is similar between wild type and *slx-1* mutants (Figure 7B, 7E, 7F). These data suggest that there is a pathway not associated with ZHP-3 foci to make a crossover in *slx-1* mutants. Whether this pathway leads to crossover formation at the center region of the chromosomes and whether these ZHP-3 foci-independent crossovers depend on MUS-81, which is known to make ZHP-3 foci-independent crossovers when there is an excess of DSBs [57], are issues that remain to be solved.

In yeast, the crossover/noncrossover decision is made very early, prior to or during the formation of stable single-end invasion (SEI) intermediates, and therefore earlier than dHJ formation [59]. Once a dHJ is formed, it is usually converted into a crossover in yeast meiosis. However, it is not known whether this tendency is conserved in other organisms. We hypothesize that DSBs introduced at the mid-section of chromosomes are converted into a noncrossover product either via a synthesis-dependent strand annealing (SDSA) pathway or via symmetric resolution of a dHJ in wild type *C. elegans*. Based on the HJ cleavage activities we observed for SLX-1, SLX-l might have a role in converting a dHJ into a noncrossover product via same sense resolution of a dHJ arising at the mid section of chromosomes during meiotic recombination (Figure 9B).

In addition to its nuclease domain, SLX-1 has a PHD finger. This type of domain is largely known to be involved in chromatin remodeling, transcriptional control and ubiquitin/SUMO E3 ligase activity. Chromosome arms, where crossovers happen at a higher frequency, are marked by methylated histone H3 lysine 9 (H3K9me) during early embryogenesis and the L3 larval stage in *C. elegans* [60], [61]. However, it is not yet known whether this kind of epigenetic mark is also observed during meiotic recombination or whether other kinds of epigenetic marks delimitate the chromosome arms and the central region in *C. elegans*. One possibility is that the PHD finger of SLX-1 is involved in epigenetic change/read and somehow divides the arms and central regions along chromosomes (Figure 9B). It will be important to investigate what kinds of epigenetic marks may be read by the PHD finger of SLX-1, and how this may regulate crossovers.

Taken together, our analysis indicates that SLX-1 is required for several kinds of mitotic DNA repair pathways and reveals a role for this protein in the regulation of meiotic crossover distribution thereby promoting the maintenance of genomic integrity. Importantly, our study leads us to propose a model in which SLX-1 functions as a noncrossover promoting factor at the crossover cold regions during meiotic recombination.

## Materials and Methods

### *C. elegans* genetics

*C. elegans* strains were cultured at 20°C under standard conditions [32]. The N2 Bristol strain was used as the wild-type background. The following mutations and chromosome rearrangements were used in this study: LGI: *slx-1(tm2644), rad-54(ok615), hT2[bli-4(e937) let-?(q782) qIs48](I; III)*; LGII: *xpf-1(e1487), dpy-10(e128), unc-4(e120), mIn1[dpy-10(e128) mIs14](II)*; LGIII: *him-18(tm2181)*, *brc-2(tm1086)*, *xnd-1(ok709), qC1[dpy-19(e1259) glp-1(q339) qIs26] (III)*; LGIV: *P_pie-1_::zhp-3::gfp, spo-11(ok79)*, *him-6(ok412), rec-8(ok978), nT1[unc-?(n754) let-? qIs50] (IV; V)*, *nT1[qIs51] (IV; V)*; LGV: *him-17(ok424), syp-2(ok307)* [16], [19], [32], [42], [62]–[67]. Transgenes: *opIs263 [Prpa-1::RPA-1-YFP::3′-UTR]* [68].

The *slx-1* allele, *tm2644*, is predicted to encode for a catalytically inactive (nuclease-negative) protein. We also tried to knockdown *slx-1* both by RNAi and by generating *slx-1(tm2644)/Df* trans-heterozygotes. RNAi utilizing feeding clones generated from either SLX-1 cDNA (Vidal ORFeome library; [69], [70]) or genomic DNA (Ahringer RNAi library; [71], [72]), did not result in depletion in either wild type or *slx-1*(*tm2644*) mutant backgrounds. Attempts to generate a *slx-1*/*Df trans*-heterozygote for classical genetic characterization of the *slx-1(tm2644)* allele were hindered because the only available deficiency encompassing that gene, *sDf4*, involves a free chromosome duplication that interferes with this analysis.

### Plasmids, cell culture, and antibodies

HIM-18 and SLX-1 open reading frames in either pENTR or pDONR derivatives were transferred to the indicated expression vectors (pDEST-myc or pDEST-HA) using the Gateway cloning system (Invitrogen) and sequence validated. 293T cells were grown in Dulbecco Modified Eagle medium (DMEM) supplemented with 10% (v/v) FBS (Invitrogen), 100 units of penicillin per ml, and 0.1 mg streptomycin per ml. Antibodies against HA (16B12; Covance) and Myc (9E10; Santa Cruz) epitopes were utilized.

### Protein interaction analysis and in vitro cleavage assays

For protein interaction studies, the indicated proteins were expressed in HEK293T cells using Lipofectamine (Invitrogen) and after 24–48 h, cells were lysed in 50 mM Tris-HCl pH 7.5, 150 mM NaCl, 0.5% NP-40, 10 mM NaF, 1 mM EDTA+protease inhibitors (ROCHE), and cleared lysates used for immunoprecipitation with the indicated antibodies. Immune complexes were washed 4–5X with lysis buffer, re-suspended in SDS laemli buffer and were subjected to polyacrylamide gel separation and immunoblotting with the indicated antibodies. Recombinant GST-h.SLX1/His-h.SLX4(SBD) was purified as previously described [5]. 5′^32P^-labeled DNA substrates (5′-flap, 3′-flap, Holliday Junction, and Replication Fork) were prepared as previously described [5]. The sequences used for the preparation of labeled substrates are presented in Table S1. Radiolabeled substrates were incubated with the indicated immune complexes expressed and purified as described above. Immune complexes were washed 3X in cleavage buffer (50 mM Tris pH 8.0, 5 mM MgCl2, 40 mM NaCl, 1 mM DTT, 100 µg/ml BSA) prior to initiating the reaction. After 30 min at 37°C, reaction mixtures were treated with 25 mM EDTA and 1% Proteinase K in 10% SDS prior to electrophoresis on either 8% polyacrylamide gels (native) or 12% polyacrylamide-urea gels (denaturing). Reaction products were visualized by autoradiography and quantified with ImageJ software.

### Time course analysis for RAD-51 foci

Quantitative analysis of RAD-51 foci was performed as in [38] except that all seven zones composing the germline were scored. 2–3 germlines were scored for each genotype. The average number of nuclei scored per zone for a given genotype was as follows, ± standard deviation: zone 1, n = 79±24; zone 2, n = 93±38; zone 3, n = 103±34; zone 4, n = 93±28; zone 5, n = 82±18; zone 6, n = 64±14; and zone 7, n = 58±13. Statistical comparisons between genotypes were performed using the two-tailed Mann-Whitney test, 95% confidence interval (C.I.).

### Measurements of RAD-51 distribution

*C. elegans* gonads were fixed and stained with rabbit α-RAD-51 (SDIX) (1∶20,000), mouse α-REC-8 (Abcam) (1∶100) and guinea pig α-HIM-8 (1∶100). Chromosome axes were traced in 3D along the REC-8 signal and straightened by using either Priism [73] or softWoRx (Applied Precision). For each chromosome axis, positions of the RAD-51 foci were measured with softWoRx. Statistical comparisons between genotypes were performed using the two-tailed Mann-Whitney test, 95% confidence interval (C.I.).

### DNA damage sensitivity experiments

To assess ionizing radiation (IR) sensitivity, animals (∼19 hours post L4 stage) were treated with 0, 50 or 100 Gy of IR from a Cs^137^ source at a dose rate of 1.86 Gy/min. For UVC sensitivity, animals were placed on the UV stratalinker 2400 (Stratagene) and exposed to 0, 300 or 600 J/m^2^. For nitrogen mustard (HN2) sensitivity, young adult animals were treated with 0, 100 or 200 µM of HN2 (mechlorethamine hydrochloride; Sigma) in M9 buffer containing *E. coli* OP50 with slow shaking in the dark for 19 hours. Treatment with camptothecin (CPT; Sigma) was similar, but with doses of 0, 500 or 1000 nM. Following treatment with IR, UVC, HN2 or CPT, animals were plated to allow recovery for 3 hours. For all damage sensitivity experiments, 21 animals were plated 7 per plate and hatching was assessed for 4 hours after the recovery. After 1.5 days, hatched worms and dead eggs were counted. Each damage condition was replicated at least three times in independent experiments.

### Quantitative analysis of germ cell apoptosis

22–24 hour post-L4 hermaphrodites were stained with acridine orange (AO) for 2 hours and mounted under coverslips in 5 µl of a 15 mM sodium azide solution on 1.5% agarose pads. Apoptotic nuclei stained with AO were observed in the late pachytene region of the germline with a Leica DM5000 B fluorescence microscope. Between 33 and 47 gonads were scored for each genotype. Statistical comparisons between genotypes were performed using the two-tailed Mann-Whitney test, 95% C.I.

### Determining crossover frequencies and distribution

Meiotic crossover frequencies were assayed utilizing single-nucleotide polymorphisms (SNP) markers as in [74], except that +/+ worms were used as a control. PCR and *Dra*I restriction digests of single worm lysates were performed as described in [75]. The following *Dra*I SNP primers were utilized: A (uCE3-637), B (CE3-127), C (snp_Y39A1), D (uCE3-1426) for chromosome III, A (uCE4-515), B (pkP4055), C (snp_F49E11), D (pkP4099) for chromosome IV, A (pkP5076), B (snp_Y61A9L), C (pkP5129), D (snp_Y17D7B) for chromosome V, and A (pkP6143), B (pkP6105), B' (snp_F11A1), C (pkP6132), D (uCE6-1554) for the X chromosome. Statistical analysis was performed using the two-tailed Fisher's Exact test, 95% C.I., as in [26] (Table S4).

Recombination analysis using visible markers was performed as in [76] and recombination frequencies were calculated as in [77].

### Yeast two-hybrid analysis

The yeast two-hybrid assay was performed according to [78]. cDNA of HIM-18 full length, SLX-1 full length, SLX-1N^1–272^ and SLX-1C^273–443^ were cloned into the Gateway donor vector (pDONR223). Each construct was then subcloned into 2 µ Gateway destination vectors pVV213 (activation domain (AD), *LEU2*+) and pVV212 (Gal4 DNA binding domain (DB), *TRP1*+). AD-Y and DB-X fusions were transformed into *MAT*a Y8800 and *MAT*a Y8930 yeast strains, respectively. These yeast strains have three reporter genes: *GAL2-ADE2*, *met2::GAL7-lacZ* and *LYS2::GAL1-HIS3*. *MAT*a Y8800 and *MAT*α Y8930 were mated on YPD plates and diploids carrying both plasmids were selected on SC-Leu-Trp plates. The interactions were assessed by growth on -His+1 mM 3-AT plates at 30°C.

## Supporting Information

Figure S1 Cleavage activity of HIM-18/SLX-1. The indicated proteins immuno-precipitated from 293T cells were incubated with ^32^P-end-labeled 5′-flap, 3′-flap, HJ, or RF substrates, and the products were separated by native gel electrophoresis and visualized by autoradiography. The labeled substrates are indicated below the gel and the * indicates the labeled strand.
(TIF)

Figure S2 Mitotic and meiotic RAD-51 foci accumulate in *slx-1*, *him-18* and *slx-1;him-18* mutants. Histograms depict the quantitation of RAD-51foci in germlines of the indicated genotypes. The number of RAD-51 foci per nucleus is categorized by the color code shown on the top. The percent of nuclei observed for each category (y-axis) are depicted for each zone along the germline axis (x-axis). 2–3 gonads were scored in each genotype. The number of RAD-51 foci per nucleus is categorized by the color code shown on the top. The percent of nuclei observed for each category (y-axis) are depicted for each zone along the germline axis (x-axis). 2–3 gonads were scored in each genotype.
(TIF)

Figure S3 The levels of RAD-51 foci are not saturated in *rad-54* mutants. Mean numbers of RAD-51 foci/nucleus are shown for *rad-54* mutants following the indicated doses of exposure. Nuclei (N) from three gonads each were scored. Error bars indicate standard error of the mean.
(TIF)

Figure S4 RPA-1-YFP foci accumulate in *brc-2 (tm1086)* mutants. (A) Representative images of RPA-1-YFP localization in RPA-1-YFP and *brc-2*; RPA-1-YFP late pachytene nuclei (zone 7). Bars, 5 µm. (B) Mean numbers of RPA-1-YFP foci/nucleus are shown. Error bars indicate standard error of the mean.
(TIF)

Figure S5 Chromosomal aberrations are not enhanced by *slx-1* mutation in *syp-2* and *rec-8* mutants. (A) High magnification images of DAPI-stained bodies in the late diakinesis oocyte just before the spermatheca (−1 oocyte). The average number of DAPI-stained bodies is shown at the bottom right of each panel. Arrowheads indicate chromosome fragments. Bar, 1 µm. (B) Average number of DAPI-stained bodies including fragments. N = number of diakinesis nuclei scored for each genotype. Bars indicate standard error. NS indicates no statistical significance. (C) Quantitation of nuclei that contain at least one chromosome fragment. (D) Quantitation of DAPI-stained bodies.
(TIF)

Table S1 DNA substrate oligonucleotides.
(XLSX)

Table S2 Number of RAD-51 foci/nucleus.
(XLSX)

Table S3 Summary of crossover distribution.
(XLSX)

Table S4 Statistical analysis of crossover distribution.
(XLSX)
