# Supplementary figures and images for: SLX-1 Is Required for Maintaining Genomic Integrity and Promoting Meiotic Noncrossovers in the Caenorhabditis elegans Germline
Source: PLoS Genet. 2012 Aug 23;8(8):e1002888. doi: 10.1371/journal.pgen.1002888 (PMC3426554; doi:10.1371/journal.pgen.1002888)

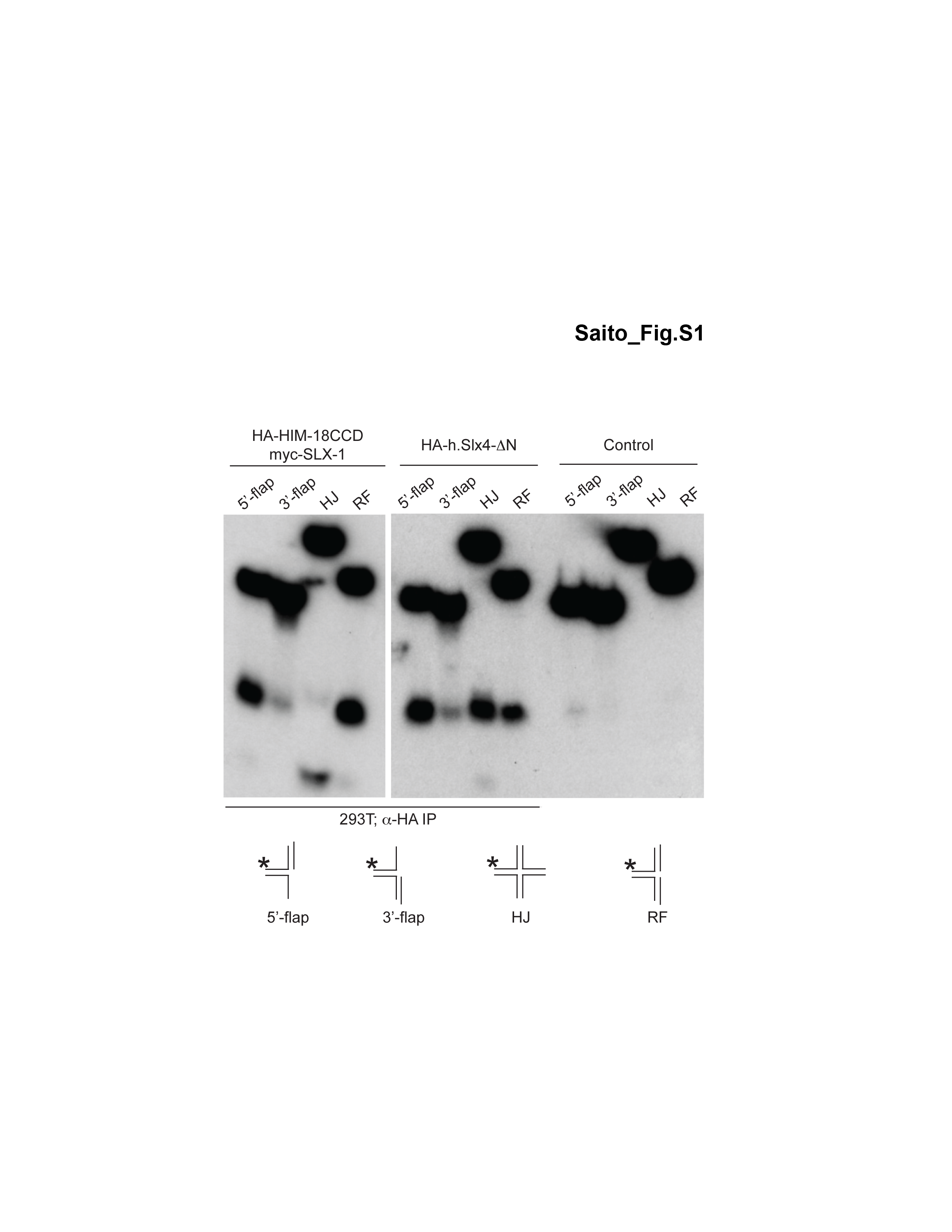

Supplement: Figure S1 — Cleavage activity of HIM-18/SLX-1. The indicated proteins immuno-precipitated from 293T cells were incubated with 32P-end-labeled 5′-flap, 3′-flap, HJ, or RF substrates, and the products were separated by native gel electrophoresis and visualized by autoradiography. The labeled substrates are indicated below the gel and the * indicates the labeled strand. (TIF) [file pgen.1002888.s001.tif]

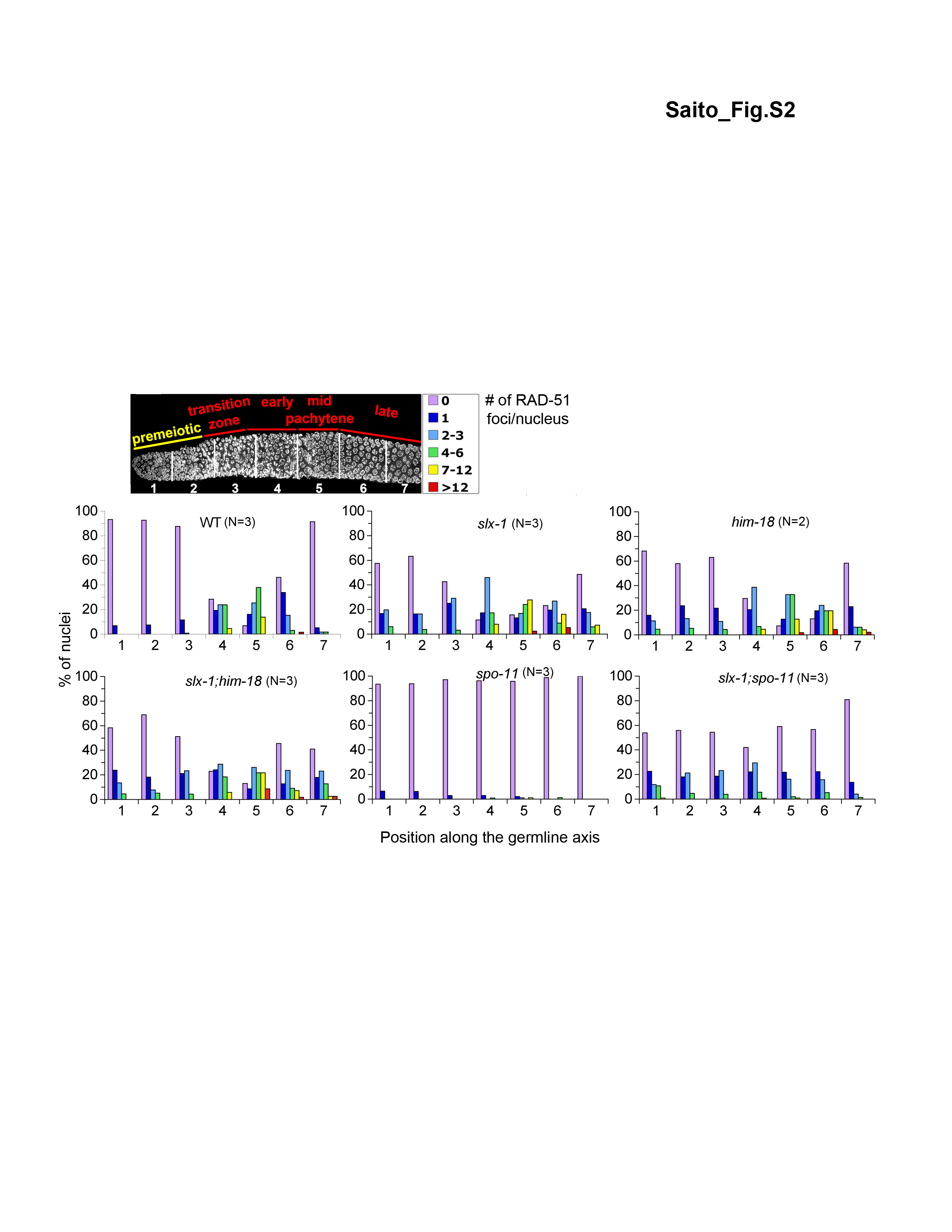

Supplement: Figure S2 — Mitotic and meiotic RAD-51 foci accumulate in slx-1, him-18 and slx-1;him-18 mutants. Histograms depict the quantitation of RAD-51foci in germlines of the indicated genotypes. The number of RAD-51 foci per nucleus is categorized by the color code shown on the top. The percent of nuclei observed for each category (y-axis) are depicted for each zone along the germline axis (x-axis). 2–3 gonads were scored in each genotype. The number of RAD-51 foci per nucleus is categorized by the color code shown on the top. The percent of nuclei observed for each category (y-axis) are depicted for each zone along the germline axis (x-axis). 2–3 gonads were scored in each genotype. (TIF) [file pgen.1002888.s002.tif]

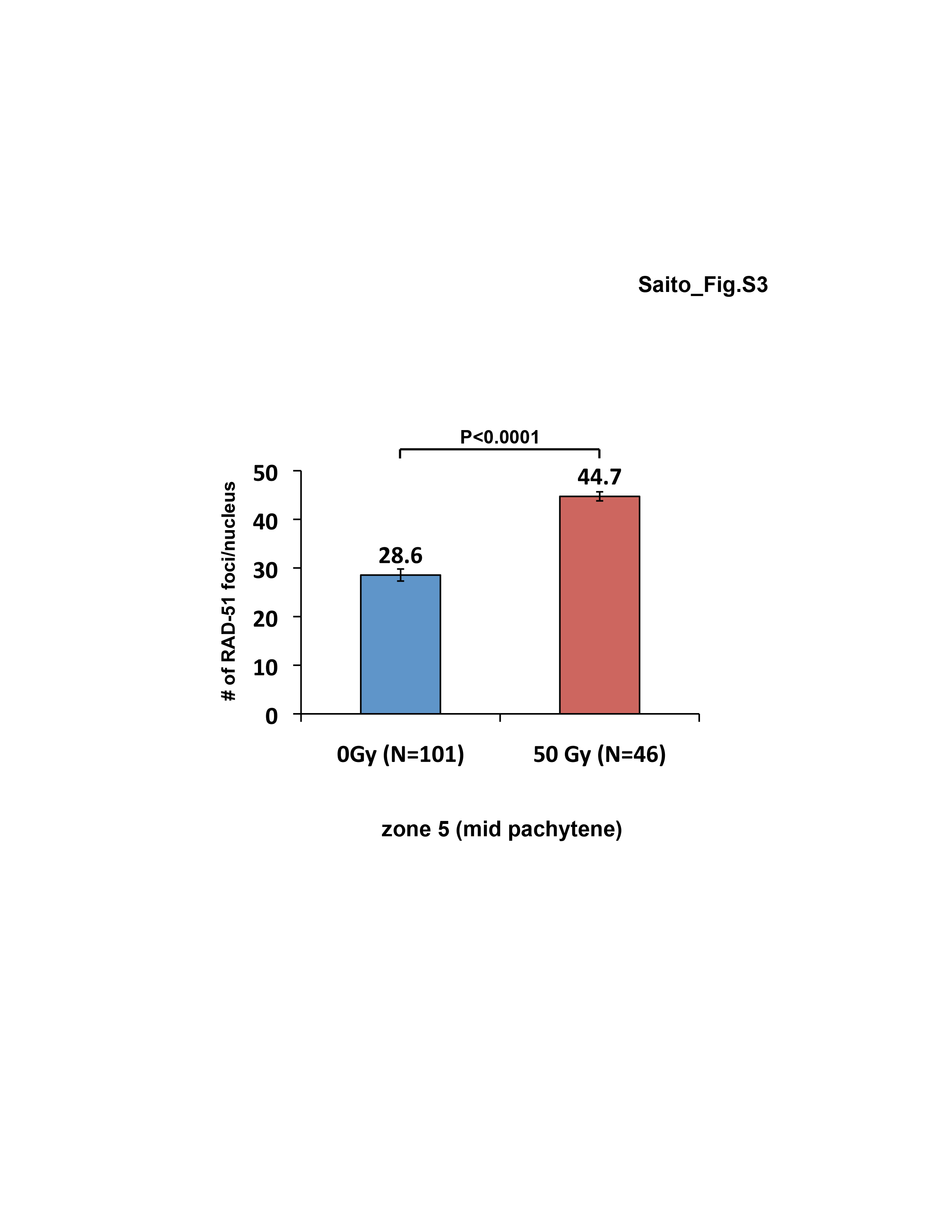

Supplement: Figure S3 — The levels of RAD-51 foci are not saturated in rad-54 mutants. Mean numbers of RAD-51 foci/nucleus are shown for rad-54 mutants following the indicated doses of exposure. Nuclei (N) from three gonads each were scored. Error bars indicate standard error of the mean. (TIF) [file pgen.1002888.s003.tif]

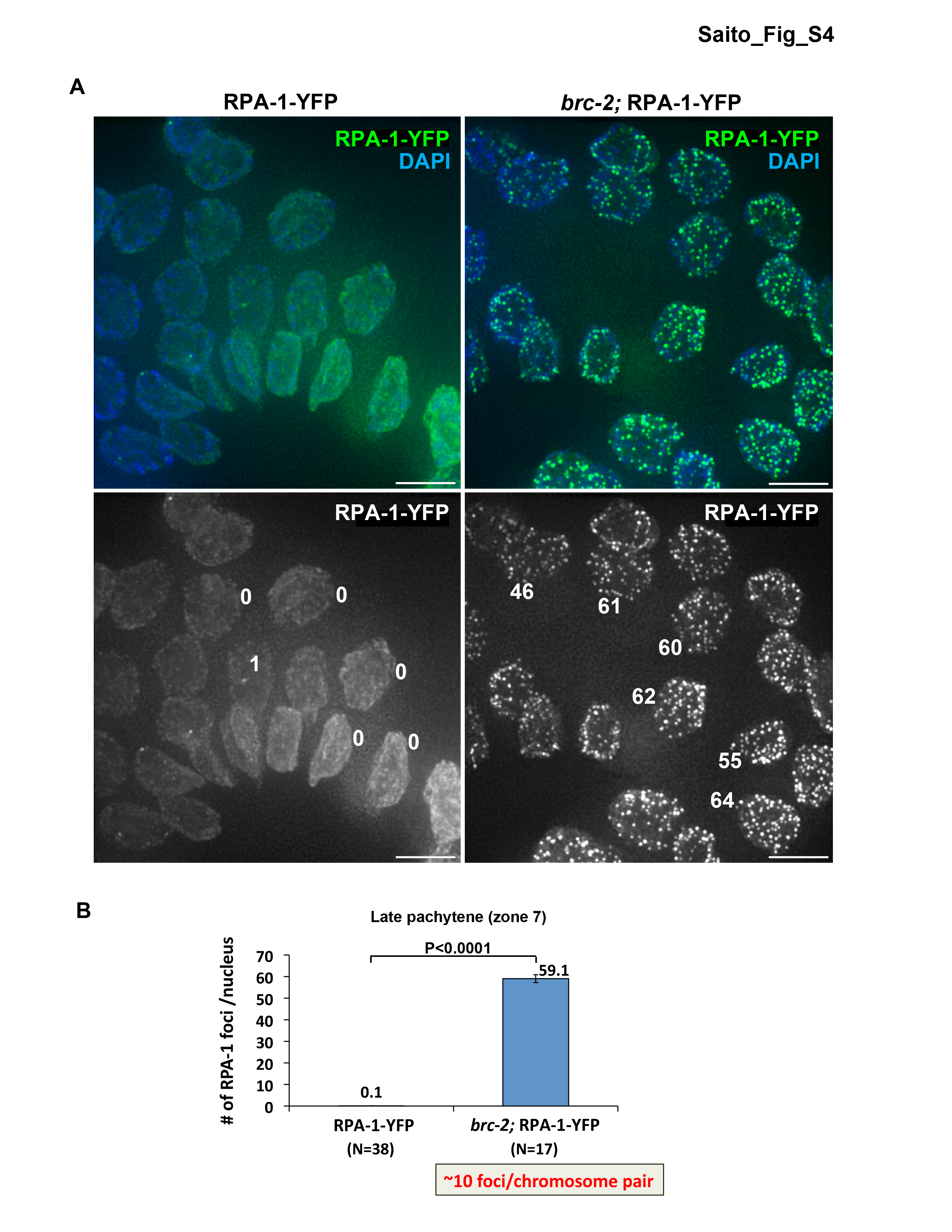

Supplement: Figure S4 — RPA-1-YFP foci accumulate in brc-2 (tm1086) mutants. (A) Representative images of RPA-1-YFP localization in RPA-1-YFP and brc-2; RPA-1-YFP late pachytene nuclei (zone 7). Bars, 5 µm. (B) Mean numbers of RPA-1-YFP foci/nucleus are shown. Error bars indicate standard error of the mean. (TIF) [file pgen.1002888.s004.tif]

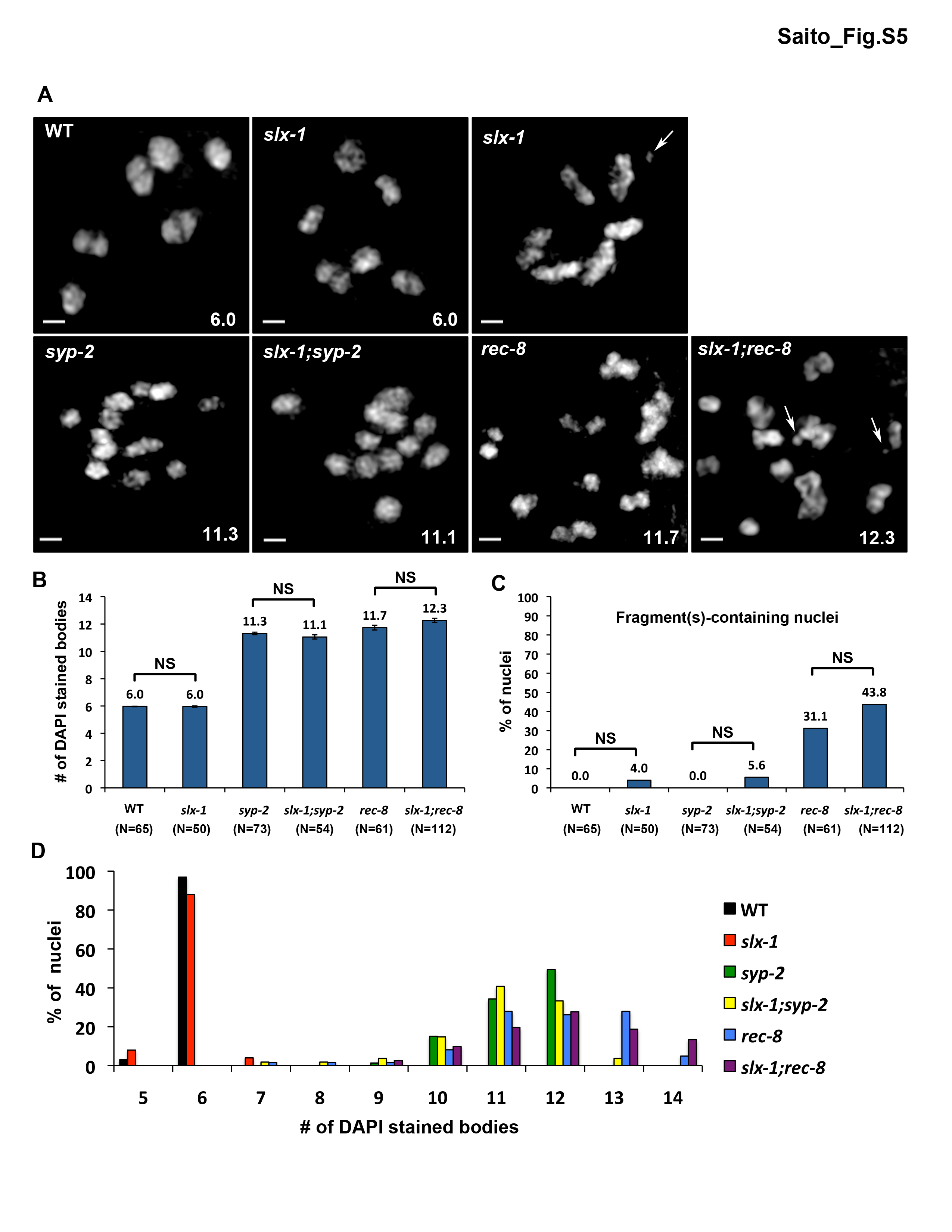

Supplement: Figure S5 — Chromosomal aberrations are not enhanced by slx-1 mutation in syp-2 and rec-8 mutants. (A) High magnification images of DAPI-stained bodies in the late diakinesis oocyte just before the spermatheca (−1 oocyte). The average number of DAPI-stained bodies is shown at the bottom right of each panel. Arrowheads indicate chromosome fragments. Bar, 1 µm. (B) Average number of DAPI-stained bodies including fragments. N = number of diakinesis nuclei scored for each genotype. Bars indicate standard error. NS indicates no statistical significance. (C) Quantitation of nuclei that contain at least one chromosome fragment. (D) Quantitation of DAPI-stained bodies. (TIF) [file pgen.1002888.s005.tif]
