# Supplementary material for: SLX-1 Is Required for Maintaining Genomic Integrity and Promoting Meiotic Noncrossovers in the Caenorhabditis elegans Germline
Source: PLoS Genet. 2012 Aug 23;8(8):e1002888. doi: 10.1371/journal.pgen.1002888 (PMC3426554; doi:10.1371/journal.pgen.1002888)
Supplement: Table S1 — DNA substrate oligonucleotides. (XLSX) [file pgen.1002888.s006.xlsx]

**Table S1. DNA substrate oligonucleotides**

| Name | Mer | Sequence |
| --- | --- | --- |
| overhang end XO-3.5 | 31 | 5'CATGGAGCTGTCTAGAGGATCCGACTATCGA |
| overhang end XO-2.5 | 31 | 5'TGGGTGAACCTGCAGGTGGGCAAAGATGTCC |
| Blunt-end XO-1 | 60 | 5'ACGCTGCCGAATTCTACCAGTGCCTTGCTAGGACATCTTTGCCCACCTGCAGGTTCACCC |
| Blunt-end XO-2 | 60 | 5'GGGTGAACCTGCAGGTGGGCAAAGATGTCCATCTGTTGTAATCGTCAAGCTTTATGCCGT |
| Blunt-end XO-3 | 60 | 5'ACGGCATAAAGCTTGACGATTACAACAGATCATGGAGCTGTCTAGAGGATCCGACTATCG |
| Blunt-end XO-4 | 60 | 5'CGATAGTCGGATCCTCTAGACAGCTCCATGTAGCAAGGCACTGGTAGAATTCGGCAGCGT |
