# Supplementary material for: SLX-1 Is Required for Maintaining Genomic Integrity and Promoting Meiotic Noncrossovers in the Caenorhabditis elegans Germline
Source: PLoS Genet. 2012 Aug 23;8(8):e1002888. doi: 10.1371/journal.pgen.1002888 (PMC3426554; doi:10.1371/journal.pgen.1002888)
Supplement: Table S2 — Number of RAD-51 foci/nucleus. (XLSX) [file pgen.1002888.s007.xlsx]

**Table S2. Number of RAD-51 foci/nucleus**

**WT**

| Zone | **1** | **2** | **3** | **4** | **5** | **6** | **7** |
| --- | --- | --- | --- | --- | --- | --- | --- |
| Ave. | 0.1 | 0.1 | 0.1 | 2.3 | 3.8 | 1.1 | 0.2 |
| N | 88 | 94 | 121 | 109 | 87 | 65 | 58 |

***slx-1***

| Zone | **1** | **2** | **3** | **4** | **5** | **6** | **7** |
| --- | --- | --- | --- | --- | --- | --- | --- |
| Ave. | 0.9 | 0.7 | 1.1 | 2.7 | 4.6 | 3.6 | 1.6 |
| N | 90 | 133 | 135 | 125 | 94 | 59 | 46 |

***rad-54(ok615)***

| Zone | **1** | **2** | **3** | **4** | **5** | **6** | **7** | **-9** | **-8** | **-7** | **-6** | **-5** | **-4** | **-3** | **-2** | **-1** |
| --- | --- | --- | --- | --- | --- | --- | --- | --- | --- | --- | --- | --- | --- | --- | --- | --- |
| Ave. | 0.8 | 1.0 | 0.9 | 5.7 | 28.6 | 48.9 | 62.9 | 76.8 | 75.8 | 72.2 | 73.5 | 64.3 | 32.5 | 12.8 | 0.2 | 0.0 |
| N | 98 | 162 | 169 | 165 | 101 | 74 | 58 | 5 | 6 | 6 | 6 | 6 | 6 | 6 | 6 | 6 |

***slx-1rad-54(ok615)***

| Zone | **1** | **2** | **3** | **4** | **5** | **6** | **7** | **-9** | **-8** | **-7** | **-6** | **-5** | **-4** | **-3** | **-2** | **-1** |
| --- | --- | --- | --- | --- | --- | --- | --- | --- | --- | --- | --- | --- | --- | --- | --- | --- |
| Ave. | 0.8 | 0.7 | 1.5 | 13.2 | 43.4 | 53.7 | 67.2 | 79.0 | 82.3 | 76.7 | 73.8 | 68.7 | 54.5 | 26.3 | 0.1 | 0.0 |
| N | 90 | 133 | 135 | 125 | 94 | 59 | 46 | 4 | 6 | 6 | 6 | 7 | 6 | 7 | 7 | 7 |

N = number of scored nuclei.
