# Supplementary material for: SLX-1 Is Required for Maintaining Genomic Integrity and Promoting Meiotic Noncrossovers in the Caenorhabditis elegans Germline
Source: PLoS Genet. 2012 Aug 23;8(8):e1002888. doi: 10.1371/journal.pgen.1002888 (PMC3426554; doi:10.1371/journal.pgen.1002888)
Supplement: Table S3 — Summary of crossover distribution. (XLSX) [file pgen.1002888.s008.xlsx]

**Table S3. Summary of crossover distribution**

| **Chromosome III** | **Left arm (A-B)** | **Center (B-C)** | **Right arm (C-D)** | **Total crossover** |
| --- | --- | --- | --- | --- |
| WT | 8 | 1 | 5 | 14 |
| *slx-1* | 8 | 4 | 6 | 18 |

| **Chromosome IV** | **Left arm (A-B)** | **Center (B-C)** | **Right arm (C-D)** | **Total crossover** |
| --- | --- | --- | --- | --- |
| WT | 30 | 5 | 12 | 47 |
| *slx-1* | 29 | 15 | 9 | 53 |
| *him-18* | 27 | 10 | 13 | 50 |
| *slx-1;him-18* | 22 | 10 | 5 | 37 |

| **Chromosome V** | **Left arm (A-B)** | **Center (B-C)** | **Right arm (C-D)** | **Total crossover** |
| --- | --- | --- | --- | --- |
| WT | 9 | 1 | 5 | 15 |
| *slx-1* | 5 | 7 | 4 | 16 |

| **Chromosome X** | **Left arm (A-B)** | **B-B’** | **B’-C** | **Right arm (C-D)** | **Total crossover** |
| --- | --- | --- | --- | --- | --- |
| WT | 39 | 29 | 13 | 38 | 119 |
| *slx-1* | 15 | 9 | 12 | 14 | 50 |
| *him-18* | 36 | 30 | 11 | 16 | 93 |
| *slx-1;him-18* | 12 | 7 | 2 | 7 | 28 |
