# Supplementary material for: SLX-1 Is Required for Maintaining Genomic Integrity and Promoting Meiotic Noncrossovers in the Caenorhabditis elegans Germline
Source: PLoS Genet. 2012 Aug 23;8(8):e1002888. doi: 10.1371/journal.pgen.1002888 (PMC3426554; doi:10.1371/journal.pgen.1002888)
Supplement: Table S4 — Statistical analysis of crossover distribution. (XLSX) [file pgen.1002888.s009.xlsx]

**Table S4. Statistical analysis of crossover distribution**

| Fisher’s exact test | *slx-1* | *him-18* | *slx-1;him-18* |
| --- | --- | --- | --- |
| Chr. III (center, others) | 0.3547 | ND | ND |
| Chr. IV (center, others) | **0.0436** | 0.2650 | 0.0833 |
| Chr. V (center, others) | **0.0373** | ND | ND |
| Chr. X (B'-C, others) | **0.0348** | 0.8312 | 0.7368 |
| Chr. X (B-C, others) | 0.4862 | 0.2048 | 0.8280 |

P-values in bold indicate statistically significant differences. ND=not determined.
